# Annotation of gene function in citrus using gene expression information and co-expression networks

**DOI:** 10.1186/1471-2229-14-186

**Published:** 2014-07-15

**Authors:** Darren CJ Wong, Crystal Sweetman, Christopher M Ford

**Affiliations:** 1 School of Agriculture, Food and Wine, University of Adelaide, Adelaide 5064, South Australia, Australia

## Abstract

**Background:**

The genus *Citrus* encompasses major cultivated plants such as sweet orange, mandarin, lemon and grapefruit, among the world’s most economically important fruit crops. With increasing volumes of transcriptomics data available for these species, Gene Co-expression Network (GCN) analysis is a viable option for predicting gene function at a genome-wide scale. GCN analysis is based on a “guilt-by-association” principle whereby genes encoding proteins involved in similar and/or related biological processes may exhibit similar expression patterns across diverse sets of experimental conditions. While bioinformatics resources such as GCN analysis are widely available for efficient gene function prediction in model plant species including *Arabidopsis,* soybean and rice, in citrus these tools are not yet developed.

**Results:**

We have constructed a comprehensive GCN for citrus inferred from 297 publicly available Affymetrix Genechip Citrus Genome microarray datasets, providing gene co-expression relationships at a genome-wide scale (33,000 transcripts). The comprehensive citrus GCN consists of a global GCN (condition-independent) and four condition-dependent GCNs that survey the sweet orange species only, all citrus fruit tissues, all citrus leaf tissues, or stress-exposed plants. All of these GCNs are clustered using genome-wide, gene-centric (guide) and graph clustering algorithms for flexibility of gene function prediction. For each putative cluster, gene ontology (GO) enrichment and gene expression specificity analyses were performed to enhance gene function, expression and regulation pattern prediction. The guide-gene approach was used to infer novel roles of genes involved in disease susceptibility and vitamin C metabolism, and graph-clustering approaches were used to investigate isoprenoid/phenylpropanoid metabolism in citrus peel, and citric acid catabolism via the GABA shunt in citrus fruit.

**Conclusions:**

Integration of citrus gene co-expression networks, functional enrichment analysis and gene expression information provide opportunities to infer gene function in citrus. We present a publicly accessible tool, Network Inference for Citrus Co-Expression (NICCE, http://citrus.adelaide.edu.au/nicce/home.aspx), for the gene co-expression analysis in citrus.

## Background

The genus *Citrus* of the plant family Rutaceae contains some of the world’s most economically important fruit crops. Major cultivated *Citrus* plants include *C. sinensis* (sweet orange), *C. reticulata* (mandarin), *C. limon* (lemon) and *C. paradisi* (grapefruit). *Citrus* species contributed to a global production of 131 million tons of fruit harvested over 8.7 million hectares in 2011 (FAOSTAT, 2013), and are primarily utilised for juice making and fresh fruit consumption. Citrus fruits contain a rich combination of nutrients important for the promotion of good health, such as simple sugars, dietary fibres, vitamins (vitamin B and C), minerals (calcium, magnesium and potassium) and bioactive phytochemicals (carotenoids, flavonoids and limonoids) [1]. The metabolic pathways by which many of these compounds are made in plants are widely known, however the genes responsible for encoding proteins of these pathways in citrus fruits remain largely undetermined.

The sequencing of plant genomes to uncover their genes, and the application of high throughput expression technologies (e.g. DNA microarray and RNA sequencing) to profile these genes, have produced large datasets of gene information and genome-scale transcriptomic data that have facilitated our understanding of many biological processes. Recently, the draft genome of sweet orange revealed that this species is highly heterozygous, with 29,445 predicted protein-coding genes out of 44,387 predicted transcripts. Of these, a total of 23,804 protein-coding genes were classified into 14,348 gene families, while the rest have been annotated as ‘hypothetical’ or ‘unknown function’ proteins [2]. Comprehensive transcriptome sequencing has also revealed insights into the molecular mechanisms underpinning key traits important for citrus fruit biology, such as vitamin C metabolism, regulation of fruit ripening and identification of disease resistance genes [2]. Taken together, these pieces of information form an invaluable resource for understanding molecular plant-pathogen interactions, abiotic stress tolerance and improvement of economically and agronomically important traits in citrus plants. However, despite recent efforts in sequencing the sweet orange genome, the majority of genes encoded in the genome remain uncharacterised, while sequencing efforts of other citrus genomes are still in progress [3].

One promising approach to improve our understanding of how these genes may function in sweet orange and related citrus plants is through Gene Co-expression Analysis (GCA). Accumulation of publicly available, genome-wide gene expression data from DNA microarrays in plants has proved useful for defining correlated expression patterns between genes using pairwise similarity metrics such as Pearson’s correlation coefficient, *r*, and subsequent genome-scale reconstruction of gene co-expression networks (GCN) [4,5]. Genes are usually represented as ‘nodes’, whilst the lines linking individual nodes, or ‘edges’, represent pairwise relationships between nodes. A collection of densely connected nodes represents a ‘cluster’ and the entire collection of nodes, edges and clusters forms the co-expression ‘network’. Often, co-expressed genes within a cluster are expected to be functionally related to genes with a similar expression pattern. This ‘guilt-by-association’ approach has become a powerful tool for transcriptional regulatory inference and understanding the evolution of transcript expression within and between plants [6,7]. Although ‘condition-independent’ GCA is common practice in plant GCA, integrating all available expression data regardless of tissue source or experimental procedure, several examples of ‘condition-dependent’ GCA have also been successfully employed to infer functions of genes in relation to conditions of interest (i.e. particular developmental stages, tissue types or stress conditions) [8-10].

To detect functional clusters (or modules) within the gene co-expression network, graph clustering and guide (or seed) gene based techniques have been successfully applied. The latter approach often requires *a priori* knowledge on function of the guide gene(s) and considers the node vicinity network of the given guide genes (i.e. genes within a defined distance, *n* from the specified guide gene) [9,11,12]. Alternatively, graph clustering algorithms such as Markov Cluster Algorithm (MCL) [13], Heuristic Cluster Chiseling Algorithm (HCCA) [14] and weighted correlation network analysis (WCGNA) [15] have been widely used to partition the complex gene co-expression network of plants in to defined functional clusters.

With emphasis on fruit crops such as sweet orange, grapevine and tomato, the application of RNA-sequencing has paved the way for transcriptome analysis of fruit crops in recent years in various stress, development and environment settings [16-22]. For the purpose of GCA, a comprehensive catalogue of experimental conditions from RNA-seq studies is still incomplete. Nevertheless, historical microarray data have provided a basis for genome-wide co-expression studies in these fruit crops [8,9,23,24]. Notably, a condition-dependent GCA coupled with a guide gene search approach was performed to identify clusters involved in biotic stress responses in citrus [23], while a combination of condition –dependent and –independent, as well as guide gene and clustering based approaches were applied to provide novel insights into grapevine berry development, photosynthesis and flavonoid metabolism [9].

Genome-wide transcript analysis studies in citrus plants including various citrus species (primarily sweet orange), tissue types and stress experiments have been widely performed on the Affymetrix Genechip Citrus Genome Array, which represents roughly 70% of the transcriptome (based on the sweet orange genome). Although these studies were mainly based on understanding a specific biological process, integration of these heterogeneous datasets for GCA can provide a functional basis for hypothesis-driven gene discovery in citrus. Here, we present a global (condition-independent) and four manually assigned (condition-dependent) GCNs of citrus inferred from 297 publicly available Affymetrix Citrus Genome Array datasets. Using genome-wide guide and graph clustering of GCNs, systematic assessments of clusters were performed using a combination of GO enrichment analysis, gene expression information and literature searches.

## Results and discussion

### General overview - Identification of biologically relevant clusters in citrus

**Figure 1:**
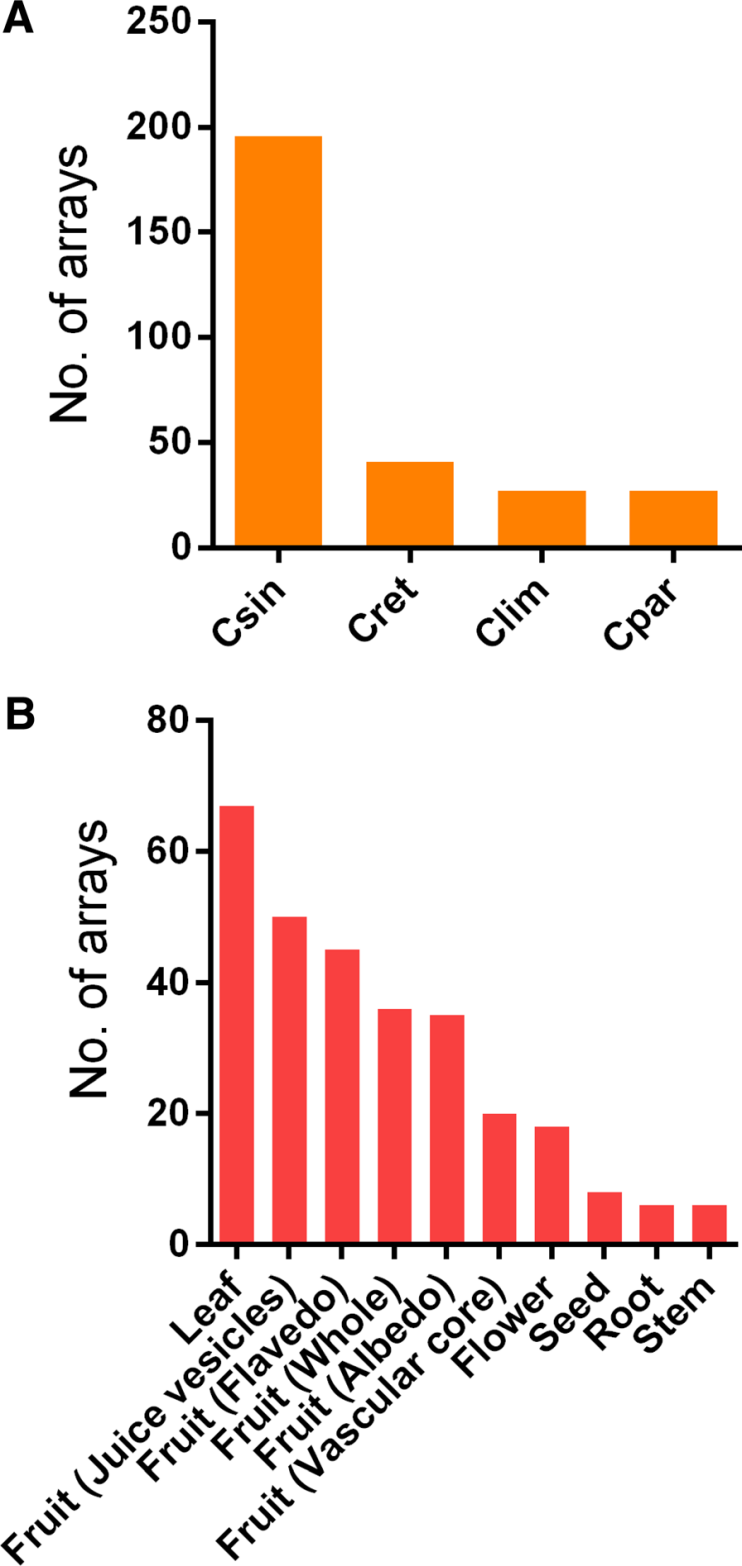
**Bar charts illustrating the classification of the citrus microarray experiments.** A total of 19 publicly available citrus microarray studies containing 297 datasets encompassing a wide range of experimental conditions and tissues were used in this study and classified according to **(A)** citrus sub-species and **(B)** organ. Additional statistics are available in Additional file 1.

A total of 297 publicly available Genechip citrus genome array datasets from 19 citrus experiments were downloaded from the NCBI gene expression omnibus repository. Descriptions pertaining to each array dataset can be found in Additional file 1: Table S1. Classification of these datasets according to sub-species, tissue type and experiment type showed that the majority of samples were from sweet orange (67%) and mandarin orange (14%), mainly from fruit (63%) and leaf (23%) tissues, and often from biotic stress treatments (66%) (Figure 1; Additional file 1: Table S2). Based on these classifications, all condition-independent datasets are referred to as ‘citrus’ while condition-dependent datasets including sweet orange–,fruit–, leaf– and stress– associated datasets will henceforth be referred to as ‘csin’, ‘fruit’, ‘leaf’ and ‘stress’, respectively. Datasets were processed and quality checked separately (see Materials and Methods). The final expression matrices from the various compendia were used to construct the condition-independent and condition-dependent co-expression networks described in this study. Correlation matrices were first calculated using all probesets (30,217) with the Pearson’s correlation coefficient (*r*) to define expression similarity between probesets. Given the difficulty in distinguishing between poorly expressed genes and background noise, and in order to provide sufficient coverage for GCA, all probesets represented on the array were included in the analysis. Given the low level of functional annotation for each probeset within the Genechip citrus genome array initially compiled by Affymetrix, the latest gene annotation of the sweet orange genome [2] was retrieved from the *Citrus sinensis* Annotation Project (CAP) [25]. The sweet orange genome annotation, which was based on evidence-based annotation and *ab initio* gene finding programs (described thoroughly in [2]), provides an accurate representation of the genes of sweet oranges. Therefore, an attempt to re-annotate the probesets was initiated. By using the consensus sequence of each probeset and performing a BLASTx search against all sweet orange protein-coding genes [2] (described in the Methods section), 23,178 probesets (from a total of 30, 217) were successfully annotated. Similarly, a separate annotation previously conducted by Zheng and Zhao [23], based on Arabidopsis orthologs and homologs managed to ascribed 22,773 probesets with a putative function. In most cases, the probesets’ annotations our’s and the latter study were similar. Nevertheless, the union of these annotations resulted in 25,147 probesets having at least one putative function ascribed to each probeset (based on either approaches), which constitutes an improvement over previous functional annotation attempts and provides a better overview of the gene function of citrus genes represented on the array. Next, raw *r* values for every relationship between probesets were transformed into highest reciprocal ranks (HRR), which serves as an index for gene co-expression. Similar to mutual ranks (MR), HRR defines the mutual co-expression relationship between two entities (genes) of interest, is relatively simple to calculate, and is robust to outliers while effectively retaining weak but significant co-expression relationships [14,26]. Statistical significance of HRR values estimated from the distribution of HRR values (of 100 microarray data permutations) [27] showed values between 310 and 340 (P < 0.01), and would provide a reasonable cut-off to infer co-expression relationships in most cases (Additional file 1: Table S3). This HRR cut-off for biological relevance value is similar to those previously reported for HRR GCN in Arabidopsis (HRR cut-off ≤ 228) [27] and grapevine (HRR cut-off ≤ 350) [9]. Additionally, HRR values ≤ 1,200 were also statistically significant (*P* < 0.05) in most cases. While this analysis revealed that HRR values ≤ 340 (and ≤ 1200) would be statistically reliable to construct the various GCNs, we empirically determined that the top 100 HRR (top *k* = 100) for each gene would also be a reasonable threshold for managing the list of co-expressed genes while maintaining biological relevance (and statistical significance). Previous studies have discussed several examples in which defining a top k^th^ threshold (i.e. top 300 MR genes) is well suited for designing a biological experiment based on co-expressed genes using the guide-gene approach [28]. The rationale of using this threshold in the present study was supported by examining the distribution of values within the top 100 HRR for each gene (Additional file 1: Table S4). The average HRR value was between 200 and 245, while the median HRR value was between 150 and 160; both of which were well under the statistical significance of HRR values at *P* < 0.01. Furthermore, HRR values at the lower bound percentile (i.e. 5^th^ and 1^st^) were statistically significant or very close to the *P* < 0.05 limit (Additional file 1: Table S4). This indicates that this threshold would be robust enough for infer meaningful co-expression relationships. Several gene co-expression studies in plants have discussed in detail the issue for defining an optimal threshold for gene co-expression, be it from raw PCC or from mutual co-expression ranks (HRR and MR), and its possible solutions [27,28]. These include defining the statistical significance of mutual co-expression ranks [27] or defining a top k^th^ threshold [28] as described earlier. In this study (and for the first time in gene co-expression studies in plants), we have leveraged these two separate approaches by showing that the top 100 HRR for each gene would be a reasonable compromise between manageability of the co-expressed genes list combined with having the statistical power in its underlying gene co-expression relationships, and therefore would be suited for downstream guide GCA inferred from the citrus dataset. Furthermore, GO enrichment analysis was then applied to functionally annotate all guide-gene co-expression clusters (30,217 clusters) and assess the predictive performance of these networks (using co-expressed genes within the top 100 HRR for each guide-gene) to recover enriched GO annotations (Table 1). As an alternative approach, when there was no previous knowledge regarding the function of the target gene, identification of densely connected modules based on the graph clustering approach was performed using MCL [13]. The MCL partitions an underlying graph based on the manipulation of transition probabilities or stochastic flows between nodes of the graph. This technique has been shown to effectively identify high-quality functional clusters and is robust to noise [29,30]. Parameter optimisation of the MCL inflation score (*I*) is often necessary to maximise clustering performance (the quality of derived GO predictions based on specificity, sensitivity and F-measure). Using this approach, we empirically determined that a threshold of HRR30 is a reasonable compromise for MCL given that increasing the threshold to HRR50 (or more) did not improve clustering performance, while reducing to HRR10 improved clustering performance slightly but excluded a greater fraction of probesets (data not shown). Similar observations have been made while determining the optimal HRR value for obtaining biologically relevant clusters [27]. Furthermore, we show that the various HRR scores (10, 20, 30, 40 and 50) used for performance evaluation and parameter optimization of MCL clustering described above were statistically significant (*P* < 2.95E-04) in all conditions defined (Additional file 1: Table S5). Similar to the guide-gene approach, an evaluation of various inflation parameters on cluster characteristics and clustering performance for each weighted HRR30 co-expression network (see Materials and Methods) was performed. We observed that an MCL *I* parameter of 1.2 and 1.3 produced the best clustering solution in terms of enrichment significance for GO biological process (BP) in most cases (Table 1). Detailed predictive performance results (F-score, specificity and sensitivity) from various methods (i.e. dataset and MCL parameters) are summarised in Additional file 1: Table S6. Using the optimal clustering solution, systematic characterisation of every module was conducted using a combination of expression data, gene ontology (GO) enrichment and literature searches. Previous co-expression studies have demonstrated that genes involved in translation, photosynthesis and phenylpropanoid metabolism were generally highly co-expressed and densely clustered across plants [27,31]. We detected several clusters of genes that were highly co-expressed across datasets and enriched with the aforementioned processes, demonstrating the robustness of the various methods for partitioning the GCNs and identifying biologically relevant clusters (Additional file 1: Table S7). These clusters may hold interesting and novel co-expression relationships and we highlight several examples of genes and clusters important for citrus fruit biology and application to the citrus industry using both guide and graph clustering approaches.

**Table 1:** Summary of citrus co-expression network features in this study

**Type**	**Dataset**	**No of nodes**	**No of edges**	**No of clusters**	**Parameter**
Guide	Citrus	30217	2322886	30217	*k* = 100
	Csin	30217	2332083	30217	*k* = 100
	Fruit	30217	2309462	30217	*k* = 100
	Leaf	30217	2288176	30217	*k* = 100
	Stress	30217	2270004	30217	*k* = 100
Graph	Citrus	25943	139370	387	*I* = 1.2
	Csin	25117	140898	940	*I* = 1.3
	Fruit	24289	136421	657	*I* = 1.2
	Leaf	25747	143925	1416	*I* = 1.3
	Stress	25964	152214	1304	*I* = 1.3

*k*, top *k* HRR for a given gene-centric cluster; *I*, MCL inflation parameter. ‘Citrus’ represents all datasets (condition-independent) used in this study while ‘Csin’, ‘Fruit’, ‘Leaf’ and ‘Stress’ represent condition-dependent datasets of sweet orange–,fruit–, leaf– and stress– only conditions respectively.

### Novel roles of Lateral organ boundaries 1 (LOB1) in citrus

Citrus bacterial canker (CBC), a disease caused by the bacteria *Xanthomonas citri* subspecies citri (Xcc), affects a wide range of citrus fruit cultivars, causing huge economic losses to the industry. A Lateral Organ Boundaries 1 (*CsLob1*) gene in sweet orange is highly induced by various Xanthomonas species [32-34] and was recently shown to function as a disease susceptibility (S) gene for CBC disease development involving both hyperplasia and hypertrophy, likely via the association with cell wall metabolic genes involved in expansion, biosynthesis and degradation [17,33]. However, the precise function and molecular targets of *Lob1* remain to be determined. To provide clues on the mode of action, co-expression analysis was carried using probesets for *Lob1*, (Cit.35190.1.S1_at and Cit.37210.1.S1_at) as guides. Using a condition-independent approach (i.e. the ‘citrus’ dataset), the top 100 genes co-expressed with *Lob1* (Cit.37210.1.S1_at) were involved in oxidative phosphorylation, ATP metabolism and cellular respiration, as well as with a few probesets likely to encode cell wall metabolism proteins. In contrast, a condition-dependent co-expression search in ‘leaf’ and ‘stress’ revealed remarkable co-expression and high enrichment of cell-wall related genes such as expansins, polygalacturonase, pectate lyase and pectin methylesterase inhibitor proteins, supporting the putative association between sweet orange *Lob1* and cell wall related enzymes (Table 2, Additional file 2: Table S1). While GO BP terms such as cell wall organisation (GO:0071555) were highly enriched in the ‘leaf’ and ‘stress’ datasets and were not unexpected, enrichment for terms involved in DNA-dependent DNA replication (GO:0006261) was interesting (Table 2, Additional file 2: Table S2). *Lob1* is also co-expressed with genes involved in DNA replication and cell cycle regulation, suggesting a novel link between cell division, cell wall metabolism and *Lob1*, which have not been associated before now. Among the co-expressed genes involved in the replication/regulation of DNA, a gene annotated as UV-B-insensitive 4 (*Uvi4*) was highly co-expressed with *Lob1. Uvi4* is a negative regulator of the anaphase-promoting complex/cyclosome, which controls cell cycle progression expression in Arabidopsis, and can cause growth defects and affect defence in plants with altered expression [35]. Therefore, abnormal cell growth (division/enlargement) during CBC disease development mediated via *Lob1* may involve the additional action of *Lob1* in increasing DNA content and affecting cell cycle-dependent expression of genes in addition to regulating cell wall metabolism, given that abnormal growth may be attributed to increased DNA content and perturbed cell cycle progression [35,36]. *Lob1* was also co-expressed with other genes, such as flavonol synthase (Cit.871.1.S1_s_at), leucoanthocyanidin dioxygenase, (Cit.5282.1.S1_at) and transcription factors (Anthocyaninless 2, Cit.7832.1.S1_at), which were involved in anthocyanin accumulation and flavonoid metabolism when restricted to the ‘fruit’ dataset (Table 2, Additional file 2: Table S1). Similar observations were made using *Lob1* (Cit.35190.1.S1_at) as a guide gene. Collectively, we demonstrate that the GCA can be leveraged to uncover various possible roles of *Lob1* in citrus. This example also demonstrated the usefulness of exploring both the condition-independent GCN ‘citrus’ and other condition-dependent GCNs for functional context, offering additional insights into BPs involved in specific physiological conditions.

**Table 2:** Guide gene co-expression analysis using LOB1 (Cit.35190.1.S1_at and Cit.37210.1.S1_at)

**Dataset**	**Enriched GO BP**	**Symbol**	**Probesets**	**P1**	**P2**
Citrus	Oxidative phophorylation (1.80E-02/2.20E-02)	ATP synthase	CIT.10573.1.S1_S_AT	ns	350
		COX2	CIT.12767.1.S1_S_AT	227	ns
		PPa1	CIT.15062.1.S1_AT	124	97
		ATP synthase	CIT.30575.1.S1_S_AT	205	279
		oxidoreductase	CIT.4015.1.S1_AT	310	ns
		oxidoreductase	CIT.4015.1.S1_S_AT	229	245
Leaf/Stress	Cell wall organisation (2.73E-05/6.45E-04)	EXPA4	CIT.102.1.S1_S_AT	27	109
Stress		EXPA4	CIT.14005.1.S1_AT	11	18
		RGP1	CIT.12232.1.S1_AT	65	75
		EXPA4	CIT.14005.1.S1_S_AT	11	23
		EXGT-A4	CIT.13455.1.S1_S_AT	100	ns
		pectinase	CIT.35756.1.S1_AT	11/34	3/8
		EXPA4	CIT.30858.1.S1_AT	10	15/191
	DNA replication (1.83E-09/ NA)	DNA primase	CIT.15305.1.S1_AT	116	ns
		ORC6	CIT.6968.1.S1_AT	133/139	ns/82
		PRL	CIT.33111.1.S1_AT	87	ns
		MCM	CIT.14761.1.S1_AT	78	ns/184
		PRL	CIT.37935.1.S1_AT	140/163	ns/104
		ATP binding	CIT.6836.1.S1_AT	66/136	131/94
		UVI4	CIT.38230.1.S1_AT	118	43/173
		MCM3	CIT.7153.1.S1_AT	47/130	ns/103
Fruit	Pigment accumulation (4.52E-04/ NA) phenylpropanoid metabolic process (2.17E-02/ NA)	ANL2	CIT.7832.1.S1_X_AT	62	ns
		ANL2	CIT.14394.1.S1_S_AT	76	ns
		GL2	CIT.28927.1.S1_AT	111	108
		ATR2	CIT.10954.1.S1_S_AT	205	ns
		FLS	CIT.871.1.S1_S_AT	39	250
		TT6	CIT.2890.1.S1_S_AT	93	124
		5MAT	CIT.14131.1.S1_AT	50	ns
		oxidoreductase	CIT.5096.1.S1_AT	104	155
		LDOX	Cit.5282.1.S1_at	137	ns
		TT7	Cit.22444.1.S1_x_at	190	150

P1 and P2 represent LOB1 probesets Cit.35190.1.S1_at and Cit.37210.1.S1_at respectively. HRR of corresponding co-expressed genes with LOB1 are shown in columns P1 and P2. For leaf and stress datasets, the HRR for each dataset are separated by a ‘/’. ns, not significant when P < 0.01.

### Vitamin C metabolism in citrus

Ascorbic acid (Ascorbate, Asc) is an efficient antioxidant, fulfilling diverse functions such as defence against oxidative and photo-oxidative stress, plant growth and development, as well as hormone and pathogen responses in plants [37]. The L-galactose pathway is by far the most prevalent and widely understood biosynthetic route of Asc, in which major controlling points of Asc biosynthesis involve the actions of GDP-mannose-3,5-epimerase (GME) and GDP-L-galactose phosphorylase/GDP-L-galactose-hexose-1-P guanylyltransferase (VTC2/5). Other pathways such as the D-galacturonate and Asc recycling pathways provide additional means of controlling Asc pools in plants [38]. To gain insights into the regulation of Asc in citrus, the gene encoding GME (Cit.23640.1.S1_s_at, Cit.7984.1.S1_s_at, Cit.7984.1.S1_at) was used in co-expression analysis. GME, the first committed enzyme of Asc biosynthesis is responsible for providing precursors (D-mannose and L-galactose) for biosynthesis of Asc and pectin network (cell wall) biogenesis [39,40]. There are two gene copies of *GME*, namely *Gme1* and *Gme2*, encoded within the sweet orange genome. Within the list of the top 100 co-expressed genes for *GME1*, were genes encoding other proteins of the primary biosynthetic pathway such as *VTC2/5* (Cit.21052.1.S1_x_at, Cit.21052.1.S1_at), *VTC1* (Cit.29407.1.S1_s_at), and *VTC4* (Cit.9252.1.S1_s_at). These were co-expressed with *GME* primarily in the leaf dataset (Table 3, Additional file 2: Table S3 - S5). Further inspection of over-represented GO terms within the co-expressed gene lists inferred from these datasets revealed that GO BP terms such as L-ascorbic acid biosynthetic process (GO:0019853,), electron transport chain (GO:0022900), and response to hormone stimulus (GO:0032870), were significantly enriched (Table 3, Additional file 2: Table S6). Conversely, when restricted to fruit only, *GME* was co-expressed with genes encoding pectinesterase and pectinesterase-inhibitors (Cit.13620.1.S1_at, Cit.17421.1.S1_at, Cit.18581.1.S1_s_at) of the D-galacturonate pathway, as well as a gene encoding monodehydroascorbate reductase (Cit.3318.1.S1_at), part of the Asc recycling pathway (Table 3, Additional file 2: Table S3 - S5). GO BP and MF terms such as cell wall organisation and pectinesterase activity were significantly enriched (FDR < 0.001) within these co-expressed genes lists (Table 3, Additional file 2: Table S6). Overall, the tissue- and condition- specificity of GME-centric clusters and their co-expressed genes were more predominant in leaf tissues and to a slight extent in citrus fruits (Additional file 2: Table S7). The coordinated expression of L-galactose pathway genes such as *GME* and *VTC2/5* in leaves is expected as it would reflect the requirement of Asc in protection against oxidative stress in actively photosynthetic tissues [41]. The lack of significant co-expression with primary Asc biosynthetic pathway genes in citrus fruits was also observed in tomato fruits [42], suggesting a lack of L-galactose pathway gene co-regulation in fruits in general. The coordination of Asc recycling as well as cell wall biogenesis and breakdown may be more relevant in contributing to Asc pools in the citrus fruit as shown in by the specific up-regulation of genes belonging to the D-galacturonate and Asc recycling pathways in fruits of strawberry [43] and grape [21,44].

**Table 3:** Guide gene co-expression analysis using GME (Cit.23640.1.S1_s_at, Cit.7984.1.S1_s_at, Cit.7984.1.S1_at)

**Dataset**	**Enriched GO BP**	**Symbol**	**Probesets**	**P1**	**P2**	**P3**
Leaf	L-ascorbic acid biosynthesis (3.00E-03/ 1.90E-02/ 7.05E-05)	VTC2	CIT.21052.1.S1_at	144	ns	ns
		VTC2	CIT.21052.1.S1_x_at	155	ns	ns
		VTC1	CIT.29407.1.S1_s_at	ns	60	169
		VTC4	CIT.9252.1.S1_s_at	ns	ns	154
	Response to hormone stimulus (6.00E-03/ 2.00E-03/ 1.10E-02)	ACT3	CIT.11614.1.S1_s_at	30	68	103
		WES1	CIT.26243.1.S1_at	94	ns	ns
		ERF13	CIT.29675.1.S1_s_at	88	ns	ns
		VH1	CIT.16607.1.S1_at	15	ns	230
		AHP1	CIT.18186.1.S1_at	155	ns	ns
		SLR	CIT.13868.1.S1_at	12	115	102
		SLR	CIT.8972.1.S1_s_at	68	10	4
		ERF7	CIT.14141.1.S1_s_at	17	99	175
		BZR1	CIT.29783.1.S1_at	ns	120	ns
		ARF8	CIT.26077.1.S1_at	ns	141	179
		BZR1	CIT.29783.1.S1_s_at	ns	178	ns
		IAA9	CIT.8966.1.S1_s_at	ns	199	ns
	Electron transport chain (6.00E-03/ 3.40E-02/ 7.05E-05)	NDHD	CIT.29311.1.S1_at	53	118	71
		NDHD	CIT.6501.1.S1_at	57	64	51
		NDHF	CIT.29293.1.S1_at	70	ns	107
		PSBE	CIT.40088.1.S1_at	27	139	89
		nsD5B	CIT.33620.1.S1_at	ns	ns	210
		COX3	CIT.18692.1.S1_at	ns	ns	224
Fruit/Citrus	Cell wall modification (NA/ 1.60E-03/ 1.30E-03) (NA/ 7.60E-04/ 3.39E-05)	pectinesterase	Cit.13620.1.S1_at	ns	16/55	27/20
		pectinesterase	Cit.17421.1.S1_at	ns	35/83	125/88
		pectinesterase	Cit.18581.1.S1_s_at	ns	115/88	191/51
		PMEPCRA	CIT.9257.1.S1_s_at	ns	ns/82	158/47
		EXPA5	CIT.2093.1.S1_s_at	ns	62	177
		COB	CIT.9596.1.S1_s_at	ns	153	202
		EXPA4	CIT.10687.1.S1_s_at	ns	151	ns
		pectinesterase	CIT.26012.1.S1_at	ns/213	ns	ns/249
	L-ascorbic acid recycling	MDAR	Cit.3318.1.S1_at	181	198	ns
Citrus	L-ascorbic acid recycling	DHAR1	Cit.31710.1.S1_s_at	ns	241	ns
		DHAR3	Cit.13490.1.S1_at	303	ns	ns
		DHAR3	Cit.13490.1.S1_x_at	203	ns	ns
		MDAR	Cit.3318.1.S1_at	181	198	ns

P1, P2 and P3 represent GME probesets Cit.23640.1.S1_s_at, Cit.7984.1.S1_s_at, and Cit.7984.1.S1_at respectively. HRR of corresponding co-expressed genes with GME are shown in columns P1, P2, P3. For fruit/citrus datasets HRR for each dataset are separated by a ‘/’. ns, not significant when P < 0.01.

### Citrus peel isoprenoid and phenylpropanoid metabolism

**Figure 2:**
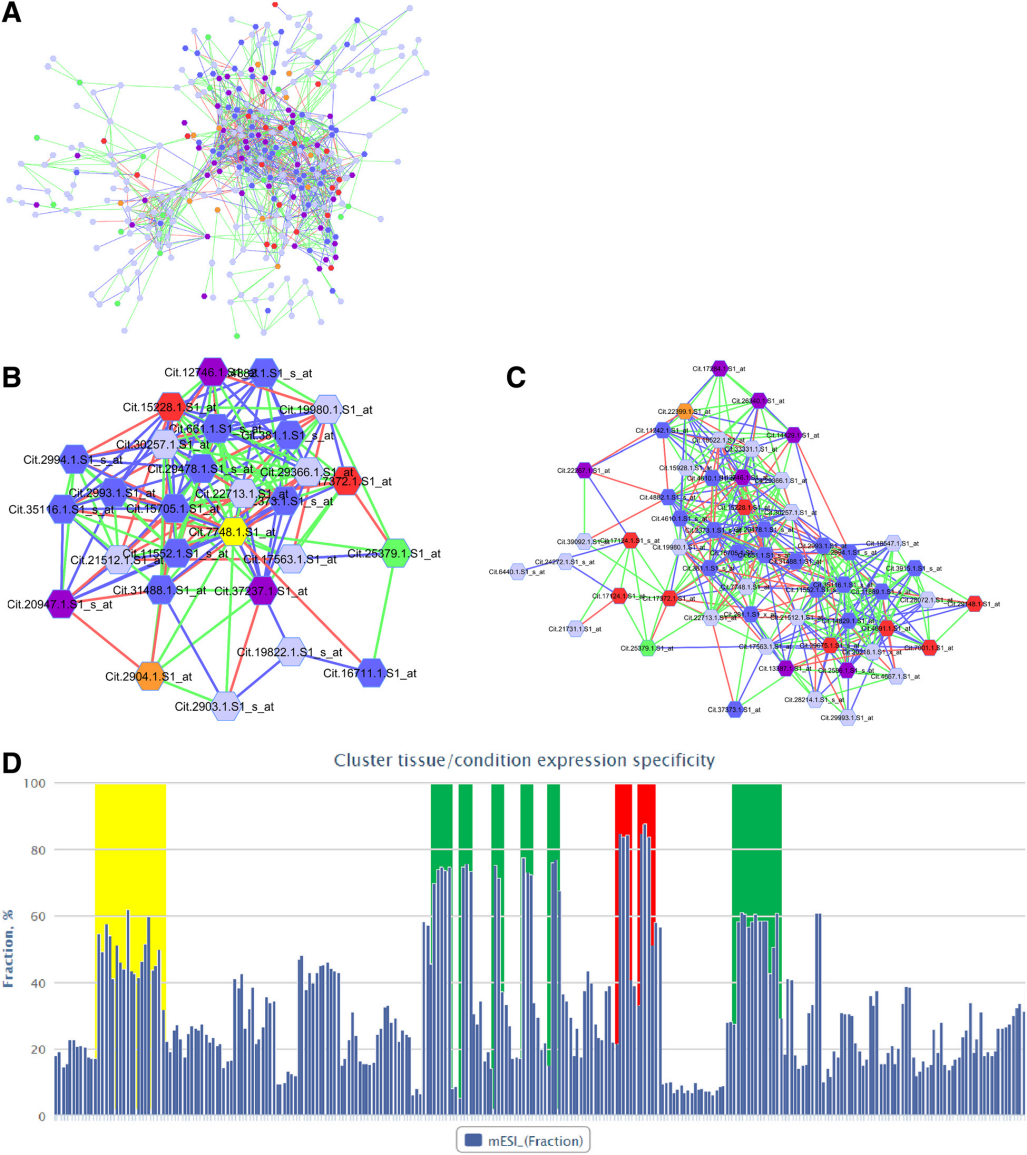
**Predicted cluster involved in citrus peel isoprenoid and phenylpropanoid metabolism (citrus_cluster14). (A)** The predicted Citrus MCL cluster 14 contained 328 nodes densely connected by 1509 edges. Genes involved in secondary metabolism (isoprenoid and phenylpropanoid), cytochrome p450/methyltransferases, lipid metabolism, hormone metabolism and signalling/transcriptional regulation were over-represented in this cluster and are coloured in purple, dark blue, orange, red and green respectively. Nodes coloured in light blue represent genes encoding proteins of miscellaneous functions (See additional files for full details). An illustration of sub-clusters for **(B)** putative zinc finger/E3 ubiquitin ligase protein (Cit.7748.1.S1_at) and **(C)** ERF13/ Ethylene response element (ERE) binding protein 1 (Cit.17124.1.S1_at, Cit.17124.1.S1_s_at, Cit.29675.1.S1_s_at, Cit.4691.1.S1_at), showing high node degree (i.e. dense connections) with many other genes within the cluster at a neighbourhood distance of 1. **(D)** Graph representation of cESI across the 297 tissues and conditions used in this study, with an expression specificity index greater than 1. Coloured boxes highlight the experimental conditions used for fruit peels (flavedo) of grapefruit (red) and sweet oranges (green), and for whole fruits of lemon (yellow).

This example will be used to demonstrate cases where graph clustering approaches can be used to infer co-expression relationships in citrus. Citrus MCL cluster 14 consisted of 328 nodes densely connected by 1,509 edges, and included many genes involved in secondary metabolism and transcriptional regulation (Figure 2A). Enriched GO parent BP terms of this module such as isoprenoid (GO:0008299) and phenylpropanoid (GO:0009699) biosynthetic process and MF terms such as oxidoreductase (GO:0016491), transferase (GO:0016740) and lyase (GO:0016829) activity were highly enriched (Table 4; Additional file 3: Table S2). Genes within the cluster were mainly involved in the biosynthesis of isoprenoid precursors (isopentenyl diphosphate and dimethylallyl diphosphate), monoterpenes, sesquiterpenes, flavanones, dihydroflavonols, anthocyanins, polymethoxylated flavones and fatty acids. Additionally, there were many genes annotated as cytochrome P450s and transferases. With no clear function in this cluster, these genes qualify as interesting candidates for gene discovery in both generalised and specialised branches of the phenylpropanoid and isoprenoid pathways in citrus. Several transcription factor/regulators belonging to the AP2/ERF, bZIP, C2H2 zinc-finger and NAM transcription factor families (among others), were densely connected to many nodes within the module (Additional file 3: Table S1). Of particular interest was a probeset annotated as a putative zinc finger/E3 ubiquitin ligase protein (Cit.7748.1.S1_at), which was highly co-expressed with genes involved in terpenoid/steroid biosynthesis such as squalene synthase 1 (SQS1; Cit.2904.1.S1_at, Cit.2903.1.S1_s_at) and mevalonate diphosphate decarboxylase (MPDC, Cit.20947.1.S1_s_at), a sterol isomerase (HYD1, Cit.17372.1.S1_at), several putative cytochrome P450s (i.e. Cit.31488.1.S1_at, Cit.15705.1.S1_at, Cit.2993.1.S1_at, Cit.29478.1.S1_s_at) and transcription factors (Cit.15228.1.S1_at, Cit.19822.1.S1_s_at) (Figure 2B, Additional file 3: Table S2). Recently an E3 ubiquitin ligase, MKB1 that was identified in *M. truncatula* and which co-expresses with triterpene saponin biosynthesis pathway genes and transcription factors, was shown to negatively regulate hydroxymethylglutaryl CoA reductase, HMGR (the main rate-limiting enzyme of the pathway) via the ubiquitin-proteasome system and thus also negatively regulate sterol and triterpene saponin biosynthesis [45]. The possibility of similar mechanisms targeting various control points of the terpenoid/steroid biosynthetic pathway could exist in other plants. Therefore, the putative zinc finger/E3 ubiquitin ligase protein (Cit.7748.1.S1_at) of citrus could be involved in the regulation of terpenoid/steroid biosynthetic pathways at other control points in citrus. Similarly, ethylene response element (ERE) binding protein 1, (ERF13; Cit.17124.1.S1_at, Cit.17124.1.S1_s_at, Cit.29675.1.S1_s_at, Cit.4691.1.S1_at) was highly co-expressed with genes involved in phenylpropanoid and flavonoid biosynthesis [i.e. Dihydroflavonol-4-reductase (DFR, Cit.28072.1.S1_at) and flavonoid 3'-hydroxylase (F3'H; Cit.4610.1.S1_at, Cit.4610.1.S1_s_at)], hormone metabolism [i.e. brassinosteroid-responsive RING-H2 (BRH; Cit.33331.1.S1_at)] as well as terpenoid metabolism [i.e. Terpene synthase 1(TPS; Cit.17284.1.S1_at) and phytoene synthase (PSY; Cit.22267.1.S1_at)] (Figure 2C, Additional file 3: Table S3). Although the molecular targets of ERF13 are yet to be elucidated, the co-expression targets of citrus ERF13 are linked to secondary metabolism. This supports the stress-and-hormone-inducible nature of ERF13, which is involved in regulation of growth and development, stress responses (biotic and abiotic), and also confers hypersensitivity to ABA in Arabidopsis [46,47].

**Table 4:** Summary of gene ontology terms enriched of citrus MCL cluster 14

**GO ID**	**GO type**	**# in input**	**FDR**	**GO description**
GO:0008299	BP	11	4.05E-06	isoprenoid biosynthesis
GO:0006721	BP	9	7.15E-05	terpenoid metabolism
GO:0009698	BP	10	3.24E-04	phenylpropanoid metabolism
GO:0016114	BP	7	7.36E-04	terpenoid biosynthesis
GO:0009813	BP	7	1.96E-03	flavonoid biosynthesis
GO:0009811	BP	5	2.97E-03	stilbene biosynthesis
GO:0010166	BP	3	3.84E-03	wax metabolism
GO:0030639	BP	5	3.92E-03	polyketide biosynthesis
GO:0009805	BP	5	3.92E-03	coumarin biosynthesis
GO:0019413	BP	5	3.92E-03	acetate biosynthesis
GO:0019438	BP	11	3.96E-03	aromatic compound biosynthesis
GO:0009809	BP	5	6.18E-03	lignin biosynthesis
GO:0046394	BP	14	8.13E-03	carboxylic acid biosynthesis
GO:0006633	BP	7	9.15E-03	fatty acid biosynthesis
GO:0009108	BP	6	2.21E-02	coenzyme biosynthesis
GO:0046356	BP	5	3.81E-02	acetyl-CoA catabolic process
GO:0009873	BP	2	4.53E-02	ethylene mediated signaling pathway
GO:0015996	BP	2	4.53E-02	chlorophyll catabolic process
GO:0016491	MF	44	5.39E-09	oxidoreductase activity
GO:0004497	MF	16	1.67E-07	monooxygenase activity
GO:0003878	MF	5	2.96E-05	ATP citrate synthase activity
GO:0016829	MF	17	2.96E-05	lyase activity
GO:0004659	MF	6	8.02E-05	prenyltransferase activity
GO:0016746	MF	13	8.02E-05	transferase activity, transferring acyl groups
GO:0004310	MF	3	8.02E-05	farnesyl-diphosphate farnesyltransferase activity
GO:0043169	MF	42	3.35E-03	cation binding
GO:0016853	MF	8	1.62E-02	isomerase activity
GO:0008171	MF	4	2.83E-02	O-methyltransferase activity
GO:0000287	MF	6	4.41E-02	magnesium ion binding
GO:0046914	MF	26	4.95E-02	transition metal ion binding

For a full list of enriched GO terms, see Additional file 3: Table S2.

Inspection of the cluster expression specificity index showed that a large fraction of genes (>70%) was specifically expressed in fruit peels (flavedo) of sweet oranges and grapefruit, but to a lesser extent in whole fruits of lemon (>50%) and with low expression specificity in leaf, flower and root tissues (Figure 2D, Additional file 3: Table S4). Significantly connected clusters 210 and 147 shared functional commonalities (enriched in secondary metabolism) as well as being enriched in other closely related biological functions such as pyruvate metabolism, glycolysis, response to oxidative stress, cytokinin biosynthesis and flower development (Additional file 3: Table S5). Overall, Citrus cluster 14 showed significant co-expression between genes involved in terpenoid and phenylpropanoid pathways, with dominant expression profiles in citrus fruit peels. This suggests that a complex regulatory network exists, underpinning the composition of secondary metabolites correlated with colour development, synthesis of phenylpropanoid derivatives and essential oil as seen in developing citrus fruits [48]. Functional evaluation of the various interesting nodes will provide the next step in the novel discovery of pathway members and regulators.

### Citric Acid Catabolism and the GABA Shunt

Citric acid is the predominant organic acid of citrus fruits. Differences in concentration of this acid in acidic and ‘acidless’ or ‘sweet’ citrus fruit species [49] may be due to regulation of citric acid catabolism [50]. The catabolism of citric acid in citrus fruits has been linked to the GABA-shunt, whereby (i) citric acid is converted to α-ketoglutaric acid via aconitase and isocitrate dehydrogenase activities (ii) α-ketoglutaric acid is converted to glutamic acid via aspartate aminotransferase or alanine aminotransferase, (iii) glutamic acid is converted to γ-aminobutyric acid (GABA) via glutamate decarboxylase, (iv) GABA is converted to succinic semialdehyde by GABA aminotransferase and (v) succinic semialdehyde is converted to succinate by succinate semialdehyde dehydrogenase, and fed back into the TCA cycle [51]. The proposed purpose of this shunt in citrus fruits is to reduce the effect of high citric acid concentrations on the pH of the fruit cell cytosol, as the biosynthesis of GABA consumes protons in the cytosol [51].

**Figure 3:**
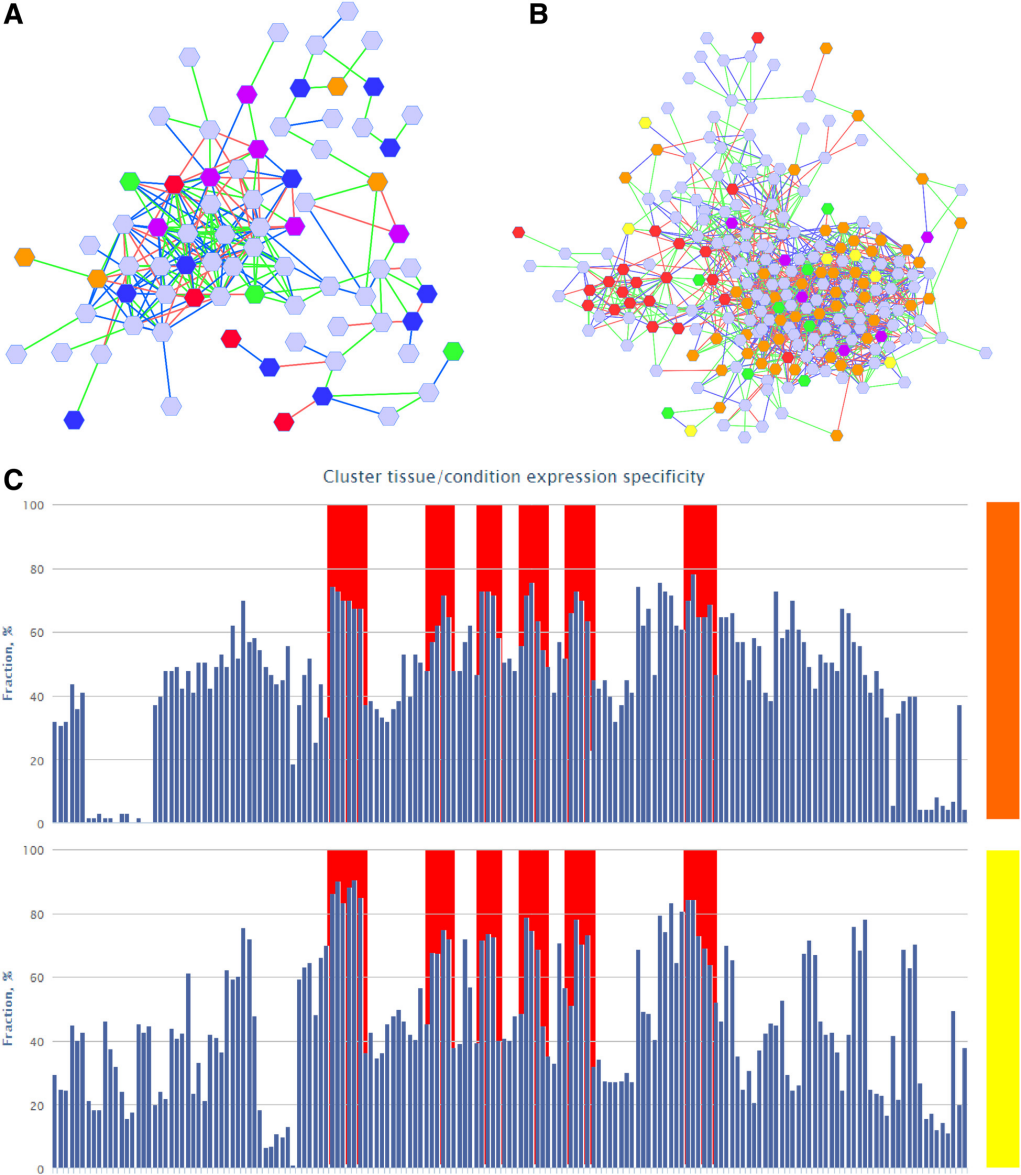
**Predicted cluster involved in GABA shunt (fruit_cluster102) and citric Acid Catabolism (fruit_cluster11). (A)** The predicted fruit MCL cluster 102 contains 76 genes connected by 242 edges. Genes involved in protein metabolism, redox, amino acid metabolism, lipid metabolism and transcriptional regulation are represented by blue, purple, red, orange and green respectively. Nodes coloured in light blue represents genes encoding proteins of miscellaneous functions/ unknown (See additional files for full details). **(B)** The predicted fruit MCL cluster 11 contains 238 genes densely connected by 1330 edges forming a central cluster. Genes involved in protein metabolism, stress, transcriptional regulation, TCA cycle/mitochondrial electron transcript and signalling are represented by orange, red, green, yellow and purple respectively. Nodes coloured in light blue represents genes encoding proteins of miscellaneous functions/ unknown (See additional files for full details). **(C)** Graph representation of cluster ESI across the 186 fruit related tissues with an expression specificity index greater than 1, in fruit MCL clusters 102 (orange bar) and 11 (yellow bar). Red boxes highlight the expression specificity of fruit-specific cluster 102 and 11 members in fruit vesicles of various sweet orange cultivars.

The fruit-specific cluster 102 (Figure 3A) contained a putative glutamate decarboxylase gene, which catalyses the proton-consuming conversion of glutamate to GABA (Cit.9469.1.S1_at), along with genes that putatively encode a pyrroline-5-carboxylate (P5C) reductase (Cit.11550.1.S1_at) and a P5C dehydrogenase (Cit.22755.1.S1), respectively catalysing the reversible conversion of proline to P5C, and the irreversible conversion of P5C to glutamic acid (Additional file 3: Table S6). This suggests that the catabolism of proline (in addition to the catabolism of citric acid), could supply glutamic acid to the GABA shunt in citrus fruits. The product of a putative ‘calcium-binding EF hand family protein’ gene also found in this cluster, may interact with citrus glutamate decarboxylase (Additional file 3: Table S7), as seen for other plant glutamate decarboxylases [for a review, see [52]]. Fruit-specific cluster 102 was connected to fruit-specific cluster 74 (connectivity score >0.03), which contained a putative NADP-dependent isocitrate dehydrogenase gene (Cit.5273.1.S1_at) that was not co-expressed with any other TCA cycle genes and may therefore be involved in citric acid catabolism and provision of α-ketoglutarate to the GABA shunt, and is likely to be controlled separately to the TCA cycle. Additionally, this cluster was enriched for glutathione dehydrogenase (ascorbate) activity (FDR 4.90E-03) (Table 5A). The catabolism of citric acid and the GABA shunt have also been associated with oxidative stress responses [53]. In addition to genes that are likely to encode enzymes of the GABA shunt as discussed above, Cluster 102 contained three dehydroascorbate reductase genes (Cit.13490.1.S1_x_at, Cit.13490.1.S1_at and Cit.23835.1.S1_s_at), a monodehydroascorbate reductase gene (Cit.3318.1.S1_at) and a thioredoxin gene (Cit.11597.1.S1_at), all of which could play a role in reactive oxygen species (ROS) scavenging. Additionally, a putative quinone reductase family protein gene that may be involved in the production of free radicals, and an NADH:ubiquinone oxidoreductase gene (Cit.14142.1.S1_at) that is likely to be involved in oxidative phosphorylation (Figure 3A) are found in this cluster.

**Table 5:** Summary of gene ontology terms enriched of fruit MCL cluster 102 (A) and 11 (B)

**GO ID**	**GO type**	**# in input**	**FDR**	**GO description**
A				
GO:0045174	MF	2	4.90E-03	glutathione dehydrogenase (ascorbate) activity
GO:0016672	MF	2	4.90E-03	oxidoreductase activity, acting on sulfur group of donors, quinone or similar compound as acceptor
GO:0015038	MF	2	6.11E-03	glutathione disulfide oxidoreductase activity
GO:0015037	MF	2	6.11E-03	peptide disulfide oxidoreductase activity
B				
GO:0009408	BP	7	2.05E-02	response to heat
GO:0019296	BP	2	2.07E-02	coenzyme M metabolism
GO:0019295	BP	2	2.07E-02	coenzyme M biosynthesis
GO:0006414	BP	3	2.07E-02	translational elongation
GO:0051603	BP	8	2.92E-02	proteolysis involved in cellular protein catabolism
GO:0044257	BP	8	2.92E-02	cellular protein catabolism
GO:0006950	BP	25	2.92E-02	response to stress
GO:0051053	BP	2	4.16E-02	negative regulation of DNA metabolism
GO:0045910	BP	2	4.16E-02	negative regulation of DNA recombination
GO:0044265	BP	8	4.16E-02	cellular macromolecule catabolism
GO:0030163	BP	8	4.16E-02	protein catabolism
GO:0000018	BP	2	4.16E-02	regulation of DNA recombination
GO:0044267	BP	32	4.76E-02	cellular protein metabolism

Meanwhile, fruit-specific cluster 11 (Figure 3B) contained 213 nodes, including a GABA aminotransferase gene, which converts GABA to succinic semialdehyde in preparation for re-entry of succinate to the TCA cycle, three putative genes of glycolysis (aldolase, enolase, glucose-6-phosphate isomerase), five putative genes of the mitochondrial electron chain (two cytochrome c oxidases and three NADH:ubiquinone reductases), and 39 genes putatively involved in regulating gene transcription and protein translation, post-translational modification and degradation (Additional file 3: Table S8). Interestingly, this cluster was enriched for heat stress (FDR 0.0205) (Table 5B) among other gene ontologies, with putative heat shock proteins being represented by eighteen genes. Therefore these TCA cycle, GABA shunt and mETC genes may be responsive to environmental stresses. There was also a putative calmodulin gene (Cit.14580.1.S1_at) and a gene putatively involved in cellular Ca^2+^ sensing (Cit.12067.1.S1_s_at), suggesting the involvement of calcium in the regulation of these pathways under stress conditions, based on the highly co-expressed genes within their sub-network (Additional file 3: Table S9). Fruit-specific cluster 11, home of the predicted GABA aminotransferase gene discussed above, also contained probesets that were likely to encode ten metallothionein proteins, two manganese superoxide dismutases and two ascorbate peroxidases, all of which are involved in ROS scavenging. This cluster also contained a senescence-associated gene with a likely role in oxidative stress tolerance and genes that putatively encoded two glycolate oxidases, which produce H_2_O_2_ (Additional file 3: Table S8). Overall, the exploration of co-expression patterns between genes involved in citric acid catabolism and the GABA shunt in the context of fruit-specific MCL clusters has highlighted relationships between putative genes of these pathways and genes involved in oxidative stress responses, with specific expression in fruit vesicles in various sweet orange cultivars including Navel, Valencia and Hamlin (Figure 3C; Additional file 3: Table S10 and S11). These genes should be examined further for their roles in determining citric acid concentration in the fruits of different citrus species and in response to abiotic stress.

### Summary and future directions

We have constructed a variety of GCNs for citrus, encompassing all of the probesets represented on the Affymetrix Citrus genome array (approximately 70% of the predicted transcriptome with reference to sweet orange), using datasets from a diverse set of experimental conditions. The functional annotation of probesets was updated to include new gene information from the sweet orange genome prediction [2] and previous Arabidopsis orthology mappings [23]. The GCNs were used to evaluate several clusters (both gene-centric and graph-partitioned), for functional context with emphasis on potential biotechnological applications. The clusters evaluated generally corroborated previous reports while revealing new insights into co-expression network structures of genes and clusters. While we highlight a previous report that has utilised an integrated transcriptome comparison and GCA to dissect the GCN underlying pathogenesis of *Candidatus Liberibacter asiaticus* infection in sweet oranges (the causal agent of Huanglongbing disease, amongst the most destructive disease in citrus) [23], the current study represents the most comprehensive GCA for citrus to date, encompassing various citrus fruits, organs and experimental conditions. However, additional transcriptomics datasets involving more tissues or organ samples, under regular field settings as well as with different stress conditions (particularly abiotic) and hormone treatments are still needed to fine tune and improve the current citrus GCN. Therefore, when sufficient new publicly available citrus microarray experiments are released, incorporation of these new data into the various citrus GCN will be needed. Similarly, with the increase of RNA-sequencing applications over microarray technologies for gene expression analysis in citrus [2,19,54,55], RNA-sequencing based GCA will also be performed in future. RNA-sequencing has inherent advantages over microarray platforms, providing greater representation and quantitation of the gene transcripts, and better discrimination between isoforms or closely related sequences [56]. Furthermore, integration of cis-regulatory, miRNA, protein-DNA and protein-protein interactions will be of great interest in future as these data will ultimately enhance our understanding of genes and encoded proteins at multiple interconnected network hierarchies as seen in the model plant Arabidopsis. For example, incorporation of sweet orange predicted protein-protein interaction network data available from CAP [25] into GCNs will be the next step to improving gene function prediction of citrus. To this end, an online publicly accessible database resource called Network Inference for Citrus Co-Expression (NICCE) has been developed (http://citrus.adelaide.edu.au/nicce/home.aspx). NICCE houses GCNs inferred from both non-targeted and manually defined conditions applicable for condition-independent and condition-dependent co-expression approaches. Thus far, the main tools include the utility to search probesets matching keywords of interest (i.e. Citrus gene ID, GO ID/description), search probesets or clusters containing enriched GO terms/descriptions, and explore individual cluster and network information. Additional tools to aid interpretation of GCN such as GO enrichment analysis, network visualisation tools using CytoscapeWeb [57], column graphs and heatmaps have also been provided. Annual updates of NICCE will be performed when new publicly available microarray experiments and improved gene predictions of the sweet orange genome becomes available. Future integration of GCN inferred from accumulating RNA-sequencing datasets in the public domain into NICCE will also be considered. A comprehensive tutorial on NICCE can be found in Additional file 4 or by visiting the NICCE website. NICCE can be accessed using modern web browsers including Chrome and Firefox with Javascript enabled and Flash plug-in installed for both performance purposes and network visualisation.

## Conclusions

We have provided a comprehensive framework for GCN inference applicable to the citrus genera, and show that meaningful co-expression relationships can be obtained in these clusters. The relevant genes and clusters were supported by the co-expression network structure, functional enrichment of co-expressed genes, gene expression specificity and literature information. For this study, we include examples of genes and clusters that are biologically relevant and are of importance to the citrus industry. We also describe NICCE (Network Inference for Citrus Co-Expression, http://citrus.adelaide.edu.au/nicce/home.aspx), a user-friendly web portal equipped with comprehensive tools for citrus researchers to rapidly mine and interpret interesting co-expression relationships of genes and clusters.

## Methods

### Raw expression data and pre-processing

A total of 297 publicly available Affymetrix Genechip Citrus Genome microarray datasets measuring the transcriptional activity of approximately 33,000 transcripts (~70% transcriptome coverage) were retrieved from the Gene Expression Omnibus, NCBI. Raw CEL files were processed using RMAExpress (http://rmaexpress.bmbolstad.com/) using the default settings to compute robust multi-array average (RMA) expression values. A total of 18 potential outlier arrays that failed the probe-level, model-based quality assessment were discarded, retaining 279 arrays for further analysis. Control probesets were also removed prior to co-expression network analysis. Generalised condition-independent co-expression network analyses for citrus species were constructed using all 279 arrays. Several condition-dependent co-expression networks were also constructed separately based on their associated meta-data (classified according to their subspecies, tissue samples and experimental conditions). Finally, gene co-expression networks were generated for the generalised, sub-species-, tissue- and stress- specific datasets by applying the procedure below.

### Rank calculation and co-expression network construction

Using Pearson’s correlation coefficient (*r*) as a metric of similarity between expression values, correlation matrices were first calculated. The *r* values for all co-expressed gene pairs were transformed into ranks in ascending order of *r* for each probeset. Highest reciprocal rank (HRR) values between pair-wise probesets were calculated using formulas (1) HRR(A,B) = [max(rank(A,B), rank(B, A))] where rank(A,B) is the transformed rank of gene B according to gene A’s co-expression list and vice versa for rank(B,A) [14]. HRR are used as an index of gene co-expression and in the construction of the aforementioned gene co-expression networks. The significance of HRR for each individual network was estimated based on 100 permutations as per [27]. Genome-scale, gene-centric co-expression clusters were created by considering each gene as a ‘seed’ or ‘guide’ and all genes within the top 100 HRR for a given gene as individual clusters. This resulted in a total of 30,217 clusters (i.e. the number of probesets represented on the array), sharing potentially overlapping co-expressed genes at a genome-wide scale. Graph clustering was performed using Markov Cluster Algorithm (MCL) [13] using MCL version 12–068 (http://micans.org/mcl/) with varying inflation values, *I*, between 1.1 and 2.0, and different HRR cut-offs to identify functional clusters. HRR networks were generated using different cut-offs, where weights of 0.2, 0.067, 0.04, 0.028 and 0.022 were given cut-off HRR scores of 10, 20, 30, 40 and 50 respectively for performance evaluation. Predicted clusters with fewer than 3 probesets are often biologically meaningless and were removed.

### Evaluation of functional enrichment, cluster characteristics and clustering performance

Assessment of gene ontology (GO) term overrepresentation within a cluster was performed using BiNGO [58]. The statistical significance for all GO biological process (BP), molecular function (MF) and cellular component (CC) terms within a cluster were evaluated using the hypergeometric distribution-adjusted Benjamini & Hochberg false discovery rate (FDR) for multiple hypothesis correction. GO annotation terms were considered significant if the corrected P-value (FDR) < 0.05 and if there were at least 2 genes associated with the same annotation. Evaluation of clustering performance using MCL at various *I* values was determined by calculating the fraction of modules enriched with one annotation at FDR < 0.05 (expressed as specificity) and the fraction of annotations enriched in at least one module at FDR < 0.05 (expressed as sensitivity), having at least 2 genes associated with the enriched annotation [59]. The specificity and sensitivity values were then summarised as a functional enrichment score, the F-measure, calculated as the harmonic mean between specificity and sensitivity [(2 × Specificity × Sensitivity/(Specificity + Sensitivity)]. Probeset expression specificity was calculated according to [60] by standardising gene expression values within, and then between microarray assays. A gene was considered well and specifically expressed in the corresponding experimental condition when the probeset expression specificity index values were > 1 and > 5, respectively. Similarly, the cluster cumulative expression specificity index (cESI) was defined as the fraction of cluster members specifically expressed in a particular tissue or condition (and across all arrays) with an expression specificity index above 1, according to [9].

### Annotation of genes and visualisation of network

Previous annotations of the Citrus probesets based on homology and orthology searches against the Arabidopsis genome and NCBI best blasts hit were downloaded from Zheng and Zhao [23] and CitrusPLEX [61], and merged into a single annotation table containing reference ID (Ref_ID), reference source (Ref_Source), reference description (Ref_Desc), E-value and percentage identity (where applicable). An update to include mappings against the latest sweet orange genome annotation was also performed using local BLAST (ncbi-blast-2.2.29+) downloaded from NCBI website (ftp://ftp.ncbi.nlm.nih.gov/blast/executables/blast+/LATEST/). Briefly, consensus sequences representing each probesets (total of 30, 217) were mapped to the latest sweet orange genome annotation using BLASTx with the default setting except the E-value and identity cut-off were 1e-20 and 40% respectively. The best blast hit for this search was considered a representative sweet orange gene identifier of the underlying probesets in the array. Additionally, electronically-inferred GO assignments of the Citrus probesets using the Blast2GO pipelines [62] were downloaded from AgriGO download centre (http://bioinfo.cau.edu.cn/agriGO/download.php) prior to GO enrichment analysis in BinGO [58]. Visualisation of nodes and edge attributes were performed using a combination of features introduced in Cytoscape 2.8 [63] and CytoscapeWeb [40].

## Competing interests

The authors declare that they have no competing interests.

## Authors' contributions

DCJW conceived the study, compiled and analyzed the microarray data, performed co-expression data analysis, constructed the database platform and drafted the manuscript. CMF and CS participated in co-expression data analysis, design and coordination of the study and assisted in drafting the manuscript. All authors have read and approved the final manuscript.

## Supplementary Material

Additional file 1 **Description of Microarray datasets and associated meta-data used in the construction of the citrus co-expression network. Table S1** shows the Affymetrix Citrus Genome array datasets used in this study, including the GEO experiment ID, GEO accession ID, title, and 4 manually curated classifications describing citrus subspecies, organ, experiment type and general curated description. **Table S2** shows the classification of datasets according to citrus sub-species, organ/tissue type, experiment type and displays the number of array datasets and fraction of each classification type relative to the total. **Table S3** contains the estimated statistically significance of HRR values in various datasets at *P* < 0.01 and *P* < 0.05. **Table S4** contains the average, median and percentile distribution of HRR values within the top 100 HRR of all guide genes (30, 217 in total) in various datasets. Values highlighted in red represent HRR values below the P < 0.05 statistical significance of HRR threshold. **Table S5** contains the *P*-value significance of HRR values 10, 20, 30, 40 and 50 used for MCL graph clustering predictive performance. **Table S6** contains the detailed predictive performance results of various datasets and MCL clustering parameters). **Table S7** contains examples of clusters and related information (i.e. GO ID, GO type, GO description, MCL inflation, Condition, # in input, # in background, P-value and FDR) predicted using MCL across various datasets, containing GO Biological Processes enriched towards translation, photosynthesis and phenylpropanoid metabolism. Click here for file

Additional file 2 **Detailed information on guide GCA results of citrus LOB1 and GME.** The excel spread sheet contains 7 tables describing the analysis results from guide GCA. **Table S1** contains lists of co-expressed genes with *LOB1* (Cit.37210.1.S1_at) across citrus, csin, fruit, leaf and stress datasets highlighted in light orange, blue, purple, green and red. **Table S2** contains the global overview of enriched GO terms of *LOB1* (Cit.37210.1.S1_at) co-expressed genes across all citrus, csin, fruit, leaf and stress datasets. **Table S3 to S5** contain lists of co-expressed genes with *GME* probesets Cit.23640.1.S1_s_at, Cit.7984.1.S1_s_at and Cit.7984.1.S1_at, respectively. Similar to table S1, co-expressed genes lists derived from citrus, csin, fruit, leaf and stress datasets were highlighted in light orange, blue, purple, green and red. **Table S6** contains the global overview of enriched GO terms of *GME* (Cit.23640.1.S1_s_at, Cit.7984.1.S1_s_at and Cit.7984.1.S1_at) co-expressed genes across all citrus, csin, fruit, leaf and stress datasets. **Table S7** contains the tissue and condition expression specificity of the GME-centric cluster. Detailed descriptions of each array dataset and the corresponding ESI values for the top 6 co-expressed probesets in each dataset are shown. Click here for file

Additional file 3 **Detailed description on graph clustering results of citrus MCL cluster 14 and fruit MCL clusters 102 and 11.** The excel spread sheet contains 11 tables describing the analysis results from of citrus MCL cluster 14 and fruit MCL clusters 102 and 11. **Table S1, S6 and S8** contains lists of genes belonging to the relevant cluster and associated information such as condition, cluster ID, probeset ID, symbol, predicted function, best Arabidopsis and sweet orange gene ID match. **Table S2** contains detailed information such as GO (ID, type, description), # in input, # in background, P-value, FDR and input list of enriched GO terms of relevant clusters. **Table S3, S7 and S9** contains lists of all co-expressed gene relationships and associated interaction weights within the relevant cluster. For table S3, rows highlighted in orange and green depict co-expressed relationships of putative zinc finger/E3 ubiquitin ligase protein (Cit.7748.1.S1_at) and ERF13/ Ethylene response element (ERE) binding protein 1, (Cit.17124.1.S1_at, Cit.17124.1.S1_s_at, Cit.29675.1.S1_s_at, Cit.4691.1.S1_at) sub-cluster. For table S7, rows highlighted in blue depict co-expressed relationships of putative ‘calcium-binding EF hand family protein’ gene (Cit.18972.1.S1_at) and green highlights its co-expression relationship with glutamate decarboxylase (Cit.9469.1.S1_at). For table S9, rows highlighted in orange and red depict co-expressed relationships of a putative calmodulin gene (Cit.14580.1.S1_at) and a gene putatively involved in cellular Ca + -sensing (Cit.12067.1.S1_s_at) respectively. **Table S4, S10 and S11** contains the relevant cluster tissue/condition expression specificity tables with detailed description of each array datasets and cESI (fraction) values which have been colour-coded. **Table S5** contains information such as rank, connectivity score, and significantly connected clusters, as well as GO (type, description and FDR values) on significantly connected clusters. Click here for file

Additional file 4 **Comprehensive tutorial for GCA in citrus using NICCE.** The pdf file contains a detailed description and tutorial of NICCE and its application to discover gene function in citrus using gene expression information and co-expression networks. Click here for file
