# Supplementary material for: Annotation of gene function in citrus using gene expression information and co-expression networks
Source: BMC Plant Biol. 2014 Jul 15;14:186. doi: 10.1186/1471-2229-14-186 (PMC4108274; doi:10.1186/1471-2229-14-186)
Supplement: Additional file 4 — Comprehensive tutorial for GCA in citrus using NICCE. The pdf file contains a detailed description and tutorial of NICCE and its application to discover gene function in citrus using gene expression information and co-expression networks. [file 1471-2229-14-186-S4.pdf]

NICCE tutorial

Brief overview

NICCE houses GCNs inferred from both non-targeted and manually defined conditions applicable for condition-independent and condition-dependent co-expression approaches. Thus far, the main tools include the utility to search probesets matching keywords of interest (i.e. Citrus gene ID, GO ID/description), search probesets or clusters containing enriched GO terms/descriptions, and explore individual cluster and network information. Additional tools to aid interpretation of GCN such as GO enrichment analysis, network visualisation tools using CytoscapeWeb [43], column graphs and heatmaps have also been provided. **Instruction of how to use the service are provided in italics.**

NICCE home page

The NICCE home page contains several search forms to retrieve co-expressed genes and related information, including a keyword search, network explorer, transcription explorer and a general download section. A progress bar indicates the loading state of the home page and all NICCE webserver pages including searches.

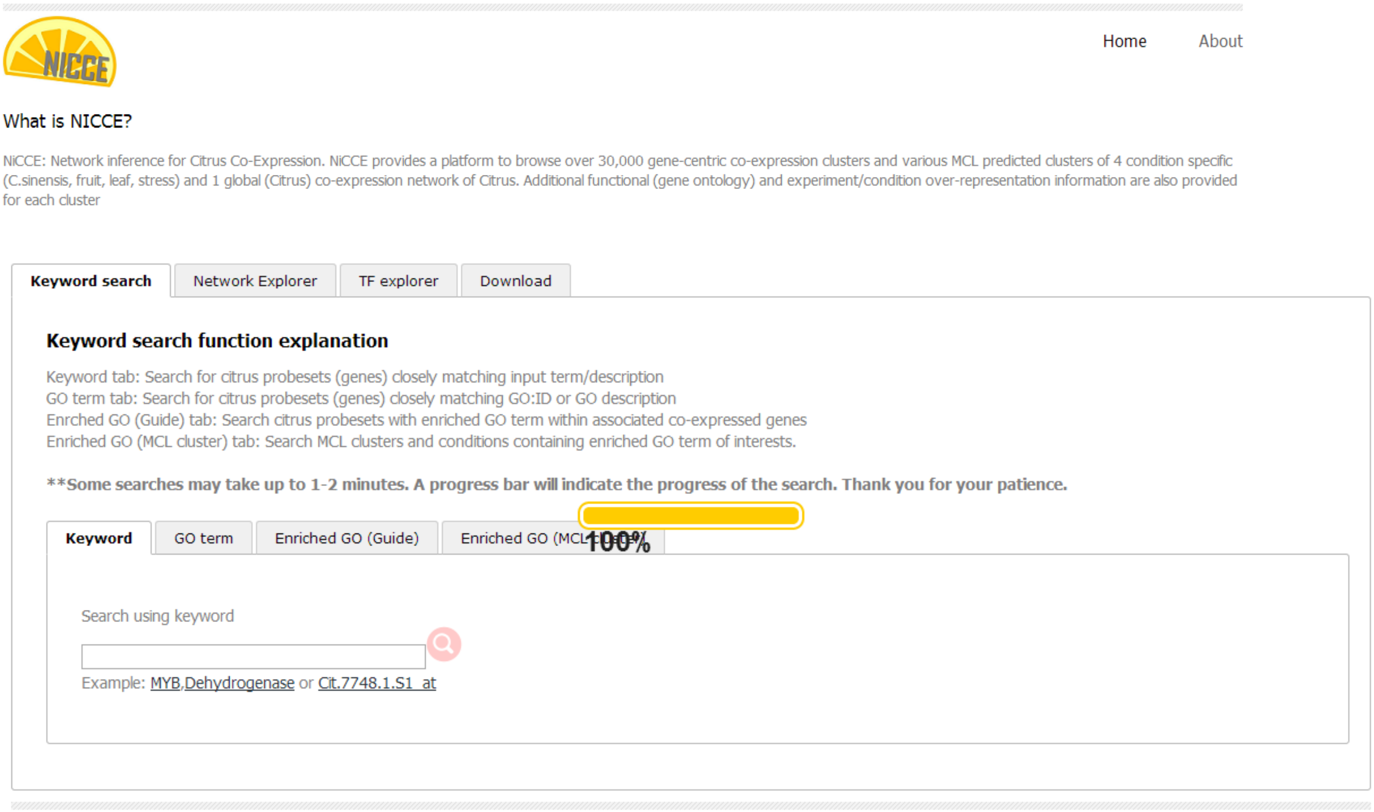

Keyword search tool: Overview

The keyword tab: Search for citrus probesets (genes) closely matching the input term or description. The GO term tab: Search for citrus probesets (genes) closely matching GO:ID or GO description. The enriched GO (Guide) tab: Search citrus probesets with enriched GO term within associated co-expressed genes. The enriched GO (MCL cluster): Search predicted MCL clusters with enriched GO:ID or GO description of interests within cluster' member genes. *After input of the relevant search terms (1 – 4), click the search icon coloured in red. Users will be diverted to the relevant keyword search page (See the* ***keyword page section*** *for more details).*

1

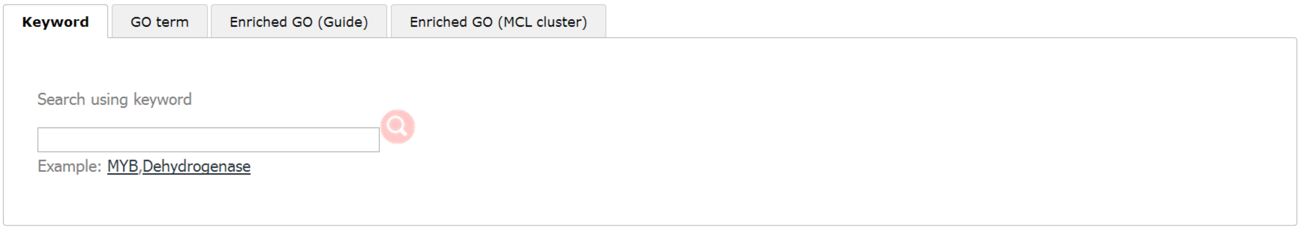

2

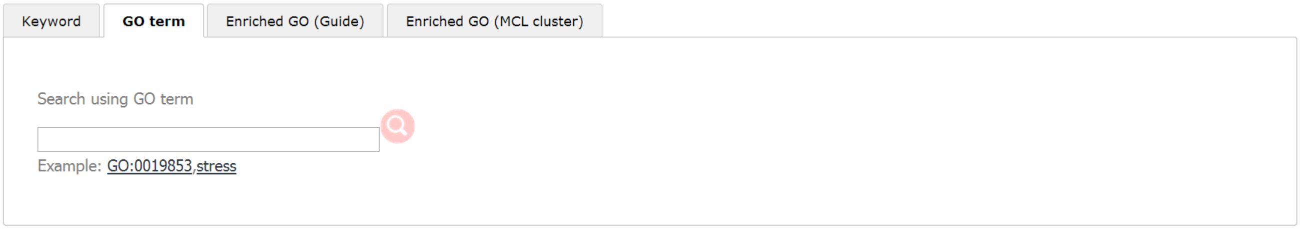

3

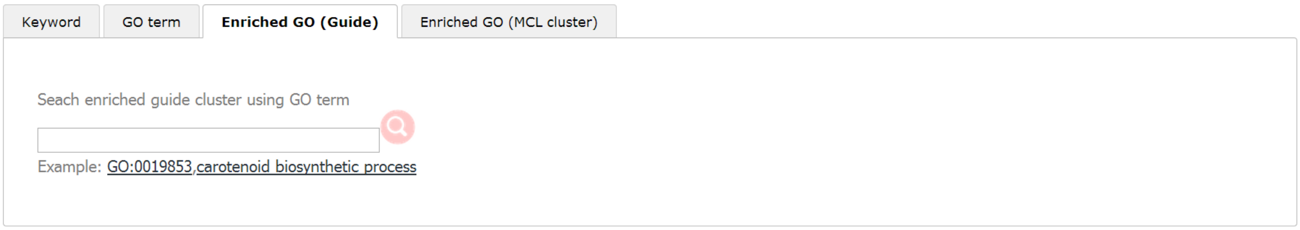

4

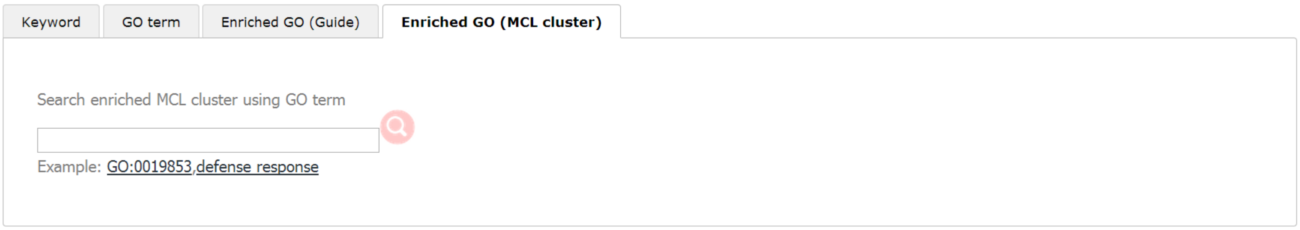

Keyword search result page: MYB as search example

The search tab contains a list of citrus probesets (genes) and relevant information closely matching the input term/description (i.e. MYB in this example). *Click on the guide column of the corresponding probesets to browse the gene-centric co-expression clusters. Click on the various MCL columns to browse MCL clusters inferred from different datasets predicted to contain the corresponding probesets.* Highlights in orange represent matches to the search input. *Click on headers to sort columns in ascending or descending order.*

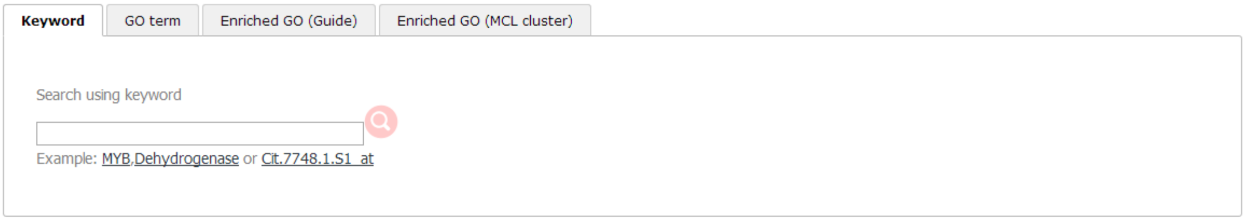

Search results

Search returned 126 matches

Lists of citrus probesets (genes) and relevant information closely matching input term/description

Guide
Click on the guide column of the corresponding probesets to browse the corresponding probesets-centric co-expression clusters
Click on the various MCL colums to browse MCL clusters inferred from different datasets predicted to contain the corresponding probesets
Highlights in orange represents matches to search input. Click on headers to sort columns in ascending or descending order

Probe	Function	Guide	Csin	Fruit	Leaf	Stress	Citrus
Cit.11186.1.S1_s_at	Function_At: myb family transcription factor<br>Function_Cs: Protein CCA1<br>Psort: NUC,CYT,PLA<br>Best At match: AT5G17300<br>Best Cs match: Cs6g16000.1	Cit.11186.1.S1_s_at	300	40	7	63	26
Cit.11247.1.S1_at	Function_At: myb family transcription factor<br>Function_Cs: Myb-like HTH transcriptional regulator-like protein<br>Psort: NUC,CHL,CYT<br>Best At match: AT2G01060<br>Best Cs match: Cs2g01490.5	Cit.11247.1.S1_at	27	70	299	217	125
Cit.11248.1.S1_s_at	Function_At: myb family transcription factor<br>Function_Cs: Myb-like HTH transcriptional regulator-like protein<br>Psort: NUC,CHL,CYT<br>Best At match: AT2G01060<br>Best Cs match: Cs2g01490.5	Cit.11248.1.S1_s_at	27	89	37	86	160
Cit.8861.1.S1_at	Function_At: MYBR1 (MYB DOMAIN PROTEIN R1)<br>Function_Cs: Tuber-specific and sucrose-responsive element binding factor (Fragment)<br>Psort: NUC,CHL,CYT<br>Best At match: AT5G67300<br>Best Cs match: Cs3g23070.1	Cit.8861.1.S1_at	0	239	1126	192	21
Cit.9262.1.S1_s_at	Function_At: myb family transcription factor<br>Function_Cs: Myb-related transcription activator, putative<br>Psort: NUC,CYT,CHL<br>Best At match: AT1G19000<br>Best Cs match: Cs5g04740.1	Cit.9262.1.S1_s_at	251	280	574	719	13
Cit.9678.1.S1_at	Function_At: transcription factor<br>Function_Cs: Alcohol dehydrogenase transcription factor myb/SANT-like protein<br>Psort: NUC,PLA,CHL<br>Best At match: AT2G44730<br>Best Cs match: Cs1g03060.1	Cit.9678.1.S1_at	179	0	0	515	39

Click to remove highlights

GO term search result page: GO:0019853 and stress as search examples

The search tab contains a list of citrus probesets (genes) closely matching GO:ID (1) or GO description (2) (i.e. GO:0019853 and stress in this example). *Click on the guide column of the corresponding probesets to browse the corresponding probesets gene-centric co-expression clusters. Click on the various MCL columns to browse MCL clusters inferred from different datasets predicted to contain the corresponding probesets.* Highlights in orange represent matches to the search input. *Click on headers to sort columns in ascending or descending order.*

Keyword | **GO term** | Enriched GO (Guide) | Enriched GO (MCL cluster)

Search using GO term

Example: GO:0019853,stress

Search results

Search returned 12 matches

List of citrus probesets (genes) closely matching GO:ID or GO description

Guide
Click on the guide column of the corresponding probesets to browse the corresponding probesets-centric co-expression clusters
Click on the various MCL colums to browse MCL clusters inferred from different datasets predicted to contain the corresponding probesets
Highlights in orange represents matches to search input. Click on headers to sort columns in ascencing or descending order

Probeset ID	GO search	Guide	Csin	Fruit	leaf	Stress	Citrus
Cit.12240.1.S1_at	GO:0019853 L-ascorbic acid biosynthetic process	Cit.12240.1.S1_at	838	67	359	109	11
Cit.13598.1.S1_s_at	GO:0019853 L-ascorbic acid biosynthetic process	Cit.13598.1.S1_s_at	167	9	256	419	124
Cit.13677.1.S1_at	GO:0019853 L-ascorbic acid biosynthetic process	Cit.13677.1.S1_at	596	37	110	15	60
Cit.19035.1.S1_at	GO:0019853 L-ascorbic acid biosynthetic process	Cit.19035.1.S1_at	560	0	424	976	0
Cit.21052.1.S1_at	GO:0019853 L-ascorbic acid biosynthetic process	Cit.21052.1.S1_at	433	28	460	213	110
Cit.21052.1.S1_x_at	GO:0019853 L-ascorbic acid biosynthetic process	Cit.21052.1.S1_x_at	433	28	460	213	110
Cit.29407.1.S1_s_at	GO:0019853 L-ascorbic acid biosynthetic process	Cit.29407.1.S1_s_at	344	66	1289	557	44
Cit.34230.1.S1_at	GO:0019853 L-ascorbic acid biosynthetic process	Cit.34230.1.S1_at	154	0	516	1234	124
Cit.6506.1.S1_at	GO:0019853 L-ascorbic acid biosynthetic process	Cit.6506.1.S1_at	392	42	293	137	246
Cit.7984.1.S1_at	GO:0019853 L-ascorbic acid biosynthetic process	Cit.7984.1.S1_at	170	0	327	511	1
Cit.7984.1.S1_s_at	GO:0019853 L-ascorbic acid biosynthetic process	Cit.7984.1.S1_s_at	170	112	327	511	124
Cit.9252.1.S1_s_at	GO:0019853 L-ascorbic acid biosynthetic process	Cit.9252.1.S1_s_at	453	32	559	564	9

Click to remove highlights

Search results

Search returned 381 matches

List of citrus probesets (genes) closely matching GO:ID or GO description

Guide
Click on the guide column of the corresponding probesets to browse the corresponding probesets-centric co-expression clusters
Click on the various MCL colums to browse MCL clusters inferred from different datasets predicted to contain the corresponding probesets
Highlights in orange represents matches to search input. Click on headers to sort columns in ascencing or descending order

Probeset ID	GO search	Guide	Csin	Fruit	leaf	Stress	Citrus
Cit.10260.1.S1_at	GO:0006950 response to stress	Cit.10260.1.S1_at	39	61	90	42	8
Cit.10260.1.S1_s_at	GO:0006950 response to stress	Cit.10260.1.S1_s_at	57	61	121	629	48
Cit.10395.1.S1_s_at	GO:0006979 response to oxidative stress	Cit.10395.1.S1_s_at	417	142	131	19	32
Cit.10542.1.S1_s_at	GO:0006950 response to stress	Cit.10542.1.S1_s_at	69	326	12	82	33
Cit.10585.1.S1_s_at	GO:0006950 response to stress	Cit.10585.1.S1_s_at	377	198	348	51	7
Cit.10665.1.S1_at	GO:0047484 regulation of response to osmotic stress	Cit.10665.1.S1_at	13	19	293	818	54
Cit.10741.1.S1_s_at	GO:0006979 response to oxidative stress	Cit.10741.1.S1_s_at	109	148	126	777	20
Cit.10835.1.S1_s_at	GO:0006979 response to oxidative stress	Cit.10835.1.S1_s_at	124	0	480	148	103
Cit.10846.1.S1_s_at	GO:0006979 response to oxidative stress	Cit.10846.1.S1_s_at	135	153	204	135	23
Cit.10909.1.S1_at	GO:0009651 response to salt stress	Cit.10909.1.S1_at	89	7	156	0	98
Cit.10910.1.S1_at	GO:0009651 response to salt stress	Cit.10910.1.S1_at	121	7	156	0	0
Cit.10912.1.S1_s_at	GO:0009651 response to salt stress	Cit.10912.1.S1_s_at	0	0	238	582	301
Cit.10919.1.S1_s_at	GO:0006950 response to stress	Cit.10919.1.S1_s_at	527	49	166	474	241
Cit.10921.1.S1_s_at	GO:0006950 response to stress	Cit.10921.1.S1_s_at	527	49	50	31	20

Click to remove highlights

Enriched GO (guide) result page: GO:0019853 and carotenoid biosynthetic process as search examples

The enriched GO (guide) search tab contains a list of citrus probesets with enriched GO terms of interest (i.e. GO:0019853 and carotenoid biosynthetic process in this example) within associated co-expressed genes (gene-centric co-expression clusters). *Click on the guide column of the corresponding probesets to browse the corresponding probesets-centric co-expression clusters.* Columns 'Csin (1)', 'Fruit (2)', 'Leaf (3)', 'Stress (4)' and 'Citrus (5)' shows the relative GO enrichment [-log10(FDR)] of co-expressed genes with corresponding probesets and heatmap visualisation of GO enrichment values whereby light red and dark red color represents no and very GO high enrichment. See colour scale below for a guide. Highlights in orange represent matches to the search input. *Click on headers to sort columns in ascending or descending order.*

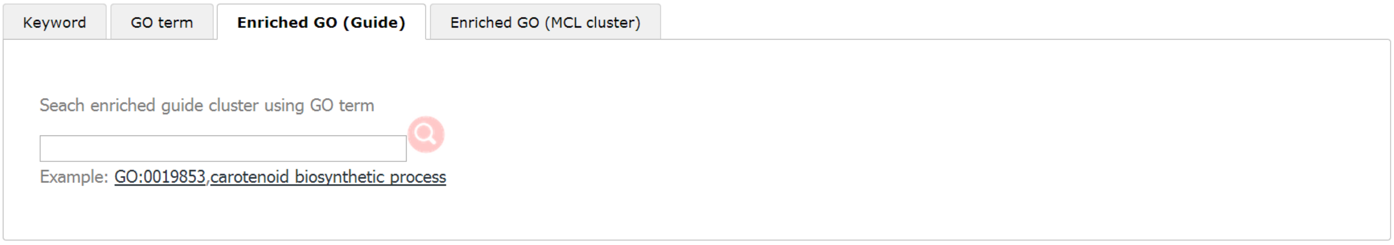

Search results

Search returned 335 matches

Lists of citrus probesets with enriched GO term of interests (from search) within associated co-expressed genes (gene-centric co-expression clusters)

Guide

Click on the guide column of the corresponding probesets to browse the corresponding probesets-centric co-expression clusters

Columns 'Csin (1)', 'Fruit (2)', 'Leaf (3)', 'Stress (4)' and 'Citrus (5)' shows the relative GO enrichment [-log10(FDR)] of co-expressed genes with corresponding probesets and heatmap visualisation of GO enrichment values whereby light red and dark red color represents no and very GO high enrichment. See color scale below for a guide

Highlights in orange represents matches to search input. Click on headers to sort columns in ascencing or descending order

GO ID	GO type	GO description	Probe	Csin	Fruit	leaf	Stress	Citrus	1	2	3	4	5
GO:0019853	BP	L-ascorbic acid biosynthetic process	Cit.19188.1.S1_at	0	0	1.42	0	0					
GO:0019853	BP	L-ascorbic acid biosynthetic process	Cit.18521.1.S1_at	0	0	0	1.43	0					
GO:0019853	BP	L-ascorbic acid biosynthetic process	Cit.18551.1.S1_at	0	0	0	0	1.5					
GO:0019853	BP	L-ascorbic acid biosynthetic process	Cit.18552.1.S1_s_at	0	0	1.42	0	0					
GO:0019853	BP	L-ascorbic acid biosynthetic process	Cit.18581.1.S1_s_at	0	1.46	0	0	0					
GO:0019853	BP	L-ascorbic acid biosynthetic process	Cit.18810.1.S1_at	0	1.32	0	0	0					
GO:0019853	BP	L-ascorbic acid biosynthetic process	Cit.18852.1.S1_at	0	0	1.72	0	0					
GO:0019853	BP	L-ascorbic acid biosynthetic process	Cit.23435.1.S1_s_at	1.31	0	0	0	0					
GO:0019853	BP	L-ascorbic acid biosynthetic process	Cit.23634.1.S1_at	1.49	0	0	0	0					
GO:0019853	BP	L-ascorbic acid biosynthetic process	Cit.23640.1.S1_s_at	1.38	0	4.15	1.37	0					
GO:0019853	BP	L-ascorbic acid biosynthetic process	Cit.2373.1.S1_s_at	0	0	1.82	0	0					
GO:0019853	BP	L-ascorbic acid biosynthetic process	Cit.22994.1.S1_x_at	1.59	0	0	0	0					
GO:0019853	BP	L-ascorbic acid biosynthetic process	Cit.21054.1.S1_at	0	0	1.4	0	0					
GO:0019853	BP	L-ascorbic acid biosynthetic process	Cit.17117.1.S1_at	0	1.74	0	1.95	1.84					
GO:0019853	BP	L-ascorbic acid biosynthetic process	Cit.2027.1.S1_s_at	0	0	0	1.96	0					
GO:0019853	BP	L-ascorbic acid biosynthetic process	Cit.20512.1.S1_x_at	0	0	1.97	0	0					
GO:0019853	BP	L-ascorbic acid biosynthetic process	Cit.20521.1.S1_at	0	0	3.88	0	0					
GO:0019853	BP	L-ascorbic acid biosynthetic process	Cit.31621.1.S1_at	0	0	2.34	0	0					
GO:0019853	BP	L-ascorbic acid biosynthetic process	Cit.3169.1.S1_s_at	1.76	0	0	1.55	0					
GO:0019853	BP	L-ascorbic acid biosynthetic process	Cit.31724.1.S1_at	0	0	1.56	1.55	0					
GO:0019853	BP	L-ascorbic acid biosynthetic process	Cit.31752.1.S1_s_at	0	0	2.3	0	0					
GO:0019853	BP	L-ascorbic acid biosynthetic process	Cit.32111.1.S1_at	0	0	1.93	2.45	0					
GO:0019853	BP	L-ascorbic acid biosynthetic process	Cit.31063.1.S1_at	0	1.32	0	0	0					

Click to remove highlights

Search results

Search returned 822 matches

Lists of citrus probesets with enriched GO term of interests (from search) within associated co-expressed genes (gene-centric co-expression clusters)

Guide

Click on the guide column of the corresponding probesets to browse the corresponding probesets-centric co-expression clusters

Columns 'Csin (1)', 'Fruit (2)', 'Leaf (3)', 'Stress (4)' and 'Citrus (5)' shows the relative GO enrichment [-log10(FDR)] of co-expressed genes with corresponding probesets and heatmap visualisation of GO enrichment values whereby light red and dark red color represents no and very GO high enrichment. See color scale below for a guide

Highlights in orange represents matches to search input. Click on headers to sort columns in ascencing or descending order

GO ID	GO type	GO description	Probe	Csin	Fruit	leaf	Stress	Citrus	1	2	3	4	5
GO:0016117	BP	carotenoid biosynthetic process	Cit.20967.1.S1_at	2.08	0	0	0	0					
GO:0016117	BP	carotenoid biosynthetic process	Cit.21634.1.S1_s_at	0	0	0	1.69	0					
GO:0016117	BP	carotenoid biosynthetic process	Cit.21606.1.S1_at	1.82	0	1.75	0	1.39					
GO:0016117	BP	carotenoid biosynthetic process	Cit.21322.1.S1_x_at	0	1.38	0	0	0					
GO:0016117	BP	carotenoid biosynthetic process	Cit.21302.1.S1_at	0	0	0	1.44	0					
GO:0016117	BP	carotenoid biosynthetic process	Cit.21291.1.S1_at	0	0	1.45	0	0					
GO:0016117	BP	carotenoid biosynthetic process	Cit.21282.1.S1_at	0	0	0	2.29	0					
GO:0016117	BP	carotenoid biosynthetic process	Cit.24660.1.S1_at	0	0	1.59	1.4	0					
GO:0016117	BP	carotenoid biosynthetic process	Cit.24656.1.S1_at	2.06	0	1.86	0	0					
GO:0016117	BP	carotenoid biosynthetic process	Cit.24615.1.S1_at	0	0	1.7	1.37	0					
GO:0016117	BP	carotenoid biosynthetic process	Cit.24522.1.S1_s_at	2.31	0	0	0	0					
GO:0016117	BP	carotenoid biosynthetic process	Cit.24418.1.S1_s_at	2.27	0	3.42	2.31	4.9					
GO:0016117	BP	carotenoid biosynthetic process	Cit.24994.1.S1_at	0	0	2.16	0	0					
GO:0016117	BP	carotenoid biosynthetic process	Cit.25815.1.S1_at	3.17	0	0	0	0					
GO:0043289	BP	apocarotenoid biosynthetic process	Cit.1105.1.S1_s_at	0	2.02	0	0	0					
GO:0016117	BP	carotenoid biosynthetic process	Cit.26078.1.S1_at	0	0	1.31	1.44	0					
GO:0016117	BP	carotenoid biosynthetic process	Cit.31286.1.S1_at	0	0	2.22	0	1.34					
GO:0016117	BP	carotenoid biosynthetic process	Cit.31190.1.S1_at	1.5	0	0	0	0					
GO:0016117	BP	carotenoid biosynthetic process	Cit.31153.1.S1_at	0	0	0	2.2	0					
GO:0016117	BP	carotenoid biosynthetic process	Cit.31557.1.S1_at	2.43	0	0	0	0					

Click to remove highlights

Enriched GO (MCL cluster) result page: GO:0019853 and defense response as search examples

The enriched GO (MCL cluster) search tab contains lists of MCL clusters and conditions containing enriched GO term of interest (i.e. GO:0019853 and defence response, in this example) and corresponding enrichment values. *Click on the various MCL columns to browse MCL clusters inferred from different datasets predicted to be enriched with GO terms of interest.* Highlights in orange represent matches to the search input. *Click on headers to sort columns in ascending or descending order.*

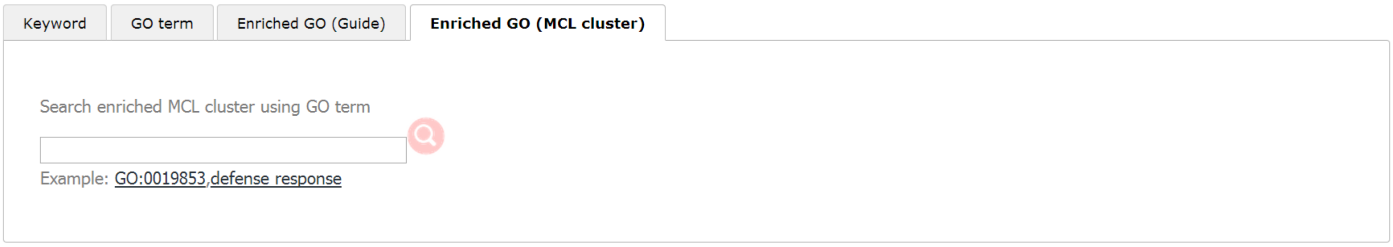

Search results

Search returned 16 matches

Lists of MCL clusters and conditions containing enriched GO term of interests and corresponding enrichment values

Guide
Click on the various MCL colums to browse MCL clusters inferred from different datasets predicted to be enriched with GO term of interests
Highlights in orange represents matches to search input. Click on headers to sort columns in ascending or descending order

GO ID	GO type	GO description	FDR	Cluster	MCL inflation	Condition
GO:0019853	BP	L-ascorbic acid biosynthetic process	0.000252	124	MCL1.2	Citrus
GO:0019853	BP	L-ascorbic acid biosynthetic process	0.0319	110	MCL1.2	Citrus
GO:0019853	BP	L-ascorbic acid biosynthetic process	0.00656	838	MCL1.3	Csin
GO:0019853	BP	L-ascorbic acid biosynthetic process	0.00729	433	MCL1.3	Csin
GO:0019853	BP	L-ascorbic acid biosynthetic process	0.0152	170	MCL1.3	Csin
GO:0019853	BP	L-ascorbic acid biosynthetic process	0.0285	560	MCL1.3	Csin
GO:0019853	BP	L-ascorbic acid biosynthetic process	0.00357	327	MCL1.3	Leaf
GO:0019853	BP	L-ascorbic acid biosynthetic process	0.00919	1289	MCL1.3	Leaf
GO:0019853	BP	L-ascorbic acid biosynthetic process	0.00938	460	MCL1.3	Leaf
GO:0019853	BP	L-ascorbic acid biosynthetic process	0.0474	559	MCL1.3	Leaf
GO:0019853	BP	L-ascorbic acid biosynthetic process	0.00381	511	MCL1.3	Stress
GO:0019853	BP	L-ascorbic acid biosynthetic process	0.00959	1234	MCL1.3	Stress
GO:0019853	BP	L-ascorbic acid biosynthetic process	0.0199	213	MCL1.3	Stress
GO:0019853	BP	L-ascorbic acid biosynthetic process	0.0296	976	MCL1.3	Stress
GO:0019853	BP	L-ascorbic acid biosynthetic process	0.0337	564	MCL1.3	Stress
GO:0019853	BP	L-ascorbic acid biosynthetic process	0.0475	557	MCL1.3	Stress

Click to remove highlights

Search results

Search returned 233 matches

Lists of MCL clusters and conditions containing enriched GO term of interests and corresponding enrichment values

Guide
Click on the various MCL colums to browse MCL clusters inferred from different datasets predicted to be enriched with GO term of interests
Highlights in orange represents matches to search input. Click on headers to sort columns in ascending or descending order

GO ID	GO type	GO description	FDR	Cluster	MCL inflation	Condition
GO:0006952	BP	defense response	7.73E-13	144	MCL1.2	Citrus
GO:0006952	BP	defense response	8.55E-06	198	MCL1.2	Citrus
GO:0050832	BP	defense response to fungus	1.15E-05	12	MCL1.2	Citrus
GO:0042742	BP	defense response to bacterium	0.000169	12	MCL1.2	Citrus
GO:0006952	BP	defense response	0.000897	12	MCL1.2	Citrus
GO:0050832	BP	defense response to fungus	0.00365	5	MCL1.2	Citrus
GO:0006952	BP	defense response	0.00515	375	MCL1.2	Citrus

Click to remove highlights

Network explorer tool: Overview

(1) *Browse MCL clusters in various condition-specific settings.* Upon coming across relevant cluster of interests and clicking, users will be diverted to the NICCE MCL cluster information page (See the **MCL cluster information page section** for more details).

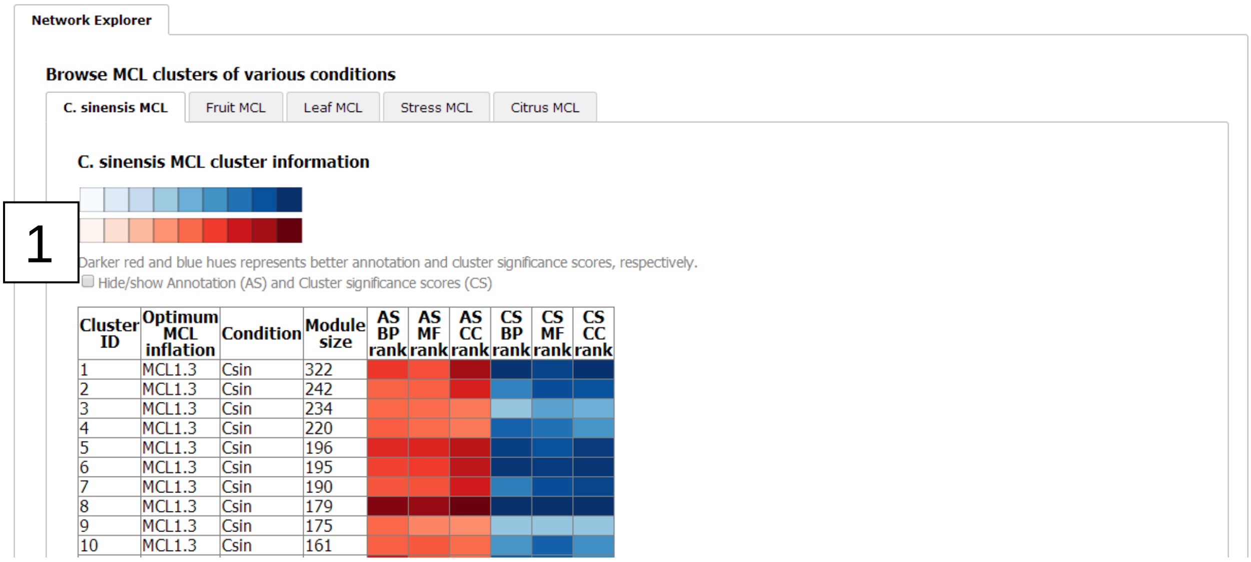

Cluster ID	Optimum MCL inflation	Condition	Module size	AS BP rank	AS MF rank	AS CC rank	CS BP rank	CS MF rank	CS CC rank
1	MCL1.3	Csin	322						
2	MCL1.3	Csin	242						
3	MCL1.3	Csin	234						
4	MCL1.3	Csin	220						
5	MCL1.3	Csin	196						
6	MCL1.3	Csin	195						
7	MCL1.3	Csin	190						
8	MCL1.3	Csin	179						
9	MCL1.3	Csin	175						
10	MCL1.3	Csin	161						

Transcription factor (TF) explorer tool: Overview

The TF explorer tab consists of a highly interactive chart showing the distribution of predicted citrus transcription factor families. *To browse the TF repertoires, simply hover the mouse cursor across the chart and a tooltip will appear showing the name of the relevant TF family and the number of matching probesets (models) predicted to contain within the array. Clicking the relevant TF family will divert users to the TF keyword search page* (See the **TF keyword page section** for more details).

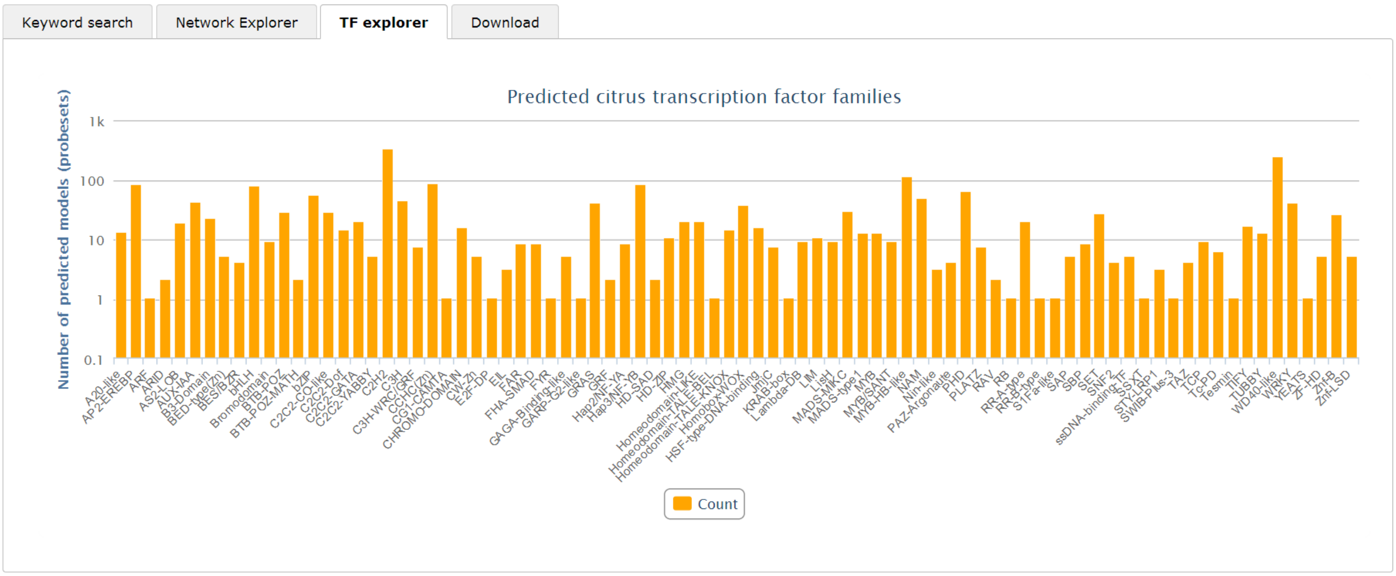

Download page: Overview

The download tab consists of all downloadable content of NICCE including annotation and experiment metadata, all condition –independent and –dependent gene co-expression networks (guide and graph clustering approach) and relevant NICCE gene co-expression network statistics.

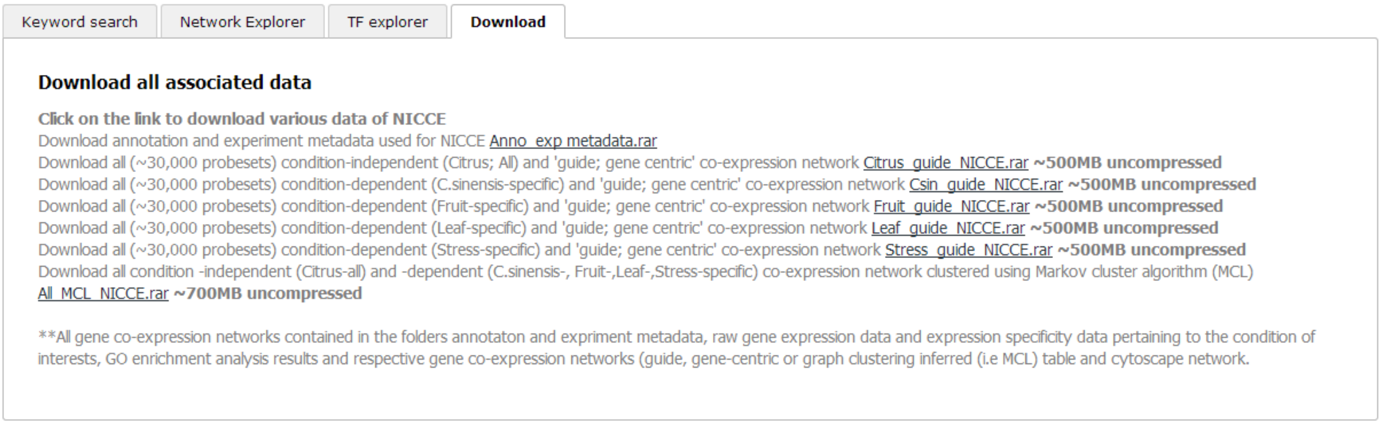

Gene-centric co-expressed cluster page: Overview

This page allows users to explore details of query gene information and tabs containing tables of the top 100 HRR ranked genes (co-expressed) with query gene, over-representation of GO terms and tissue/condition specificity graph and raw gene expression data (with heatmap illustration tool) underlying the inferred gene co-expression network in different conditions [e.g. 'Citrus (condition-independent)', 'Csin(condition-specific)', 'Fruit(condition-specific)', 'Leaf(condition-specific)' and 'Stress(condition-specific)']. Additional tabs contain the global overview of enriched GO terms of co-expression cluster (gene-centric) from all 5 datasets and MCL clusters predicted to contain the corresponding query probeset in different datasets.

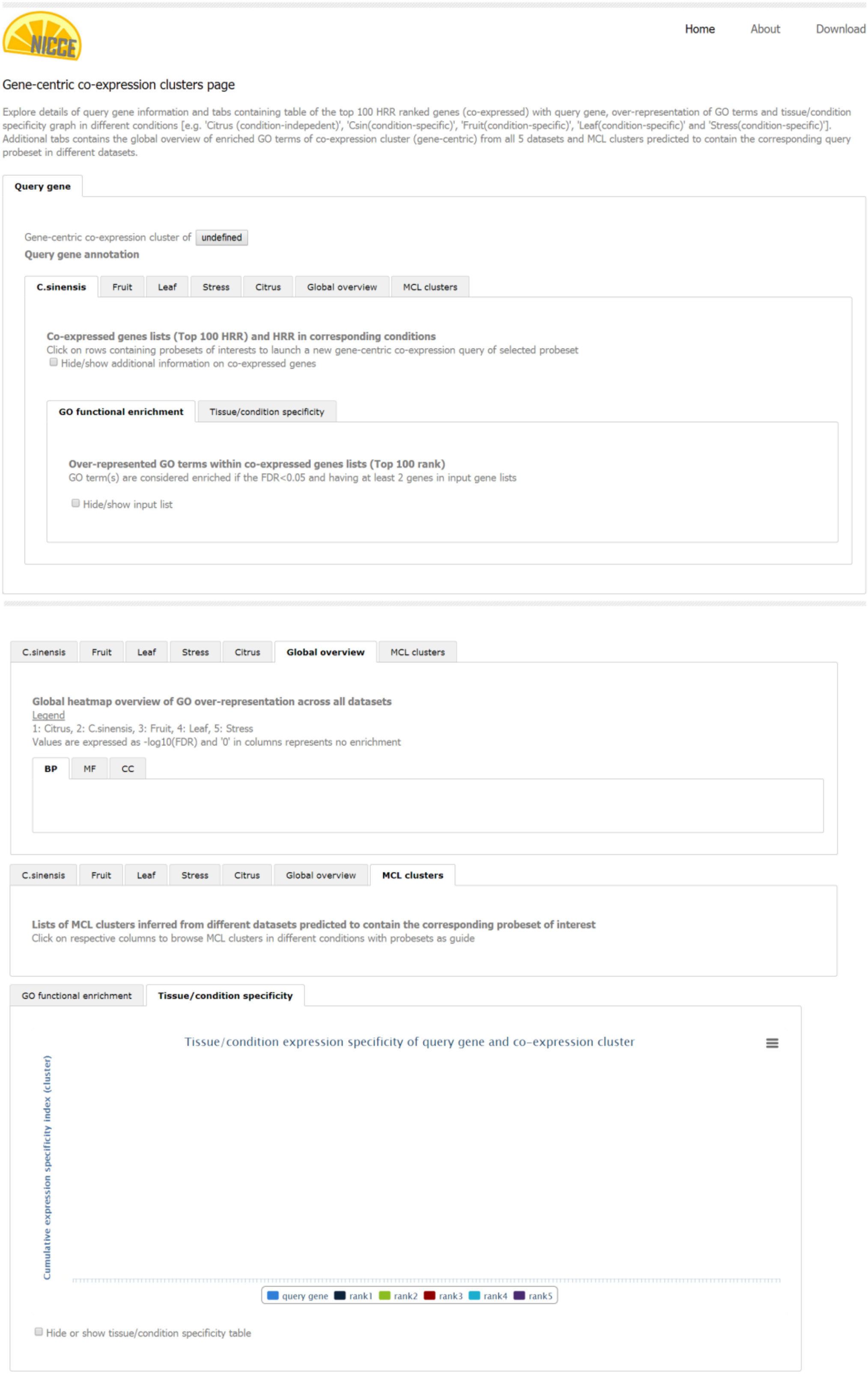

NICCE
Home
About
Download
Gene-centric co-expression clusters page
Explore details of query gene information and tabs containing table of the top 100 HRR ranked genes (co-expressed) with query gene, over-representation of GO terms and tissue/condition specificity graph in different conditions [e.g. 'Citrus (condition-indepedent)', 'Csin(condition-specific)', 'Fruit(condition-specific)', 'Leaf(condition-specific)' and 'Stress(condition-specific)']. Additional tabs contains the global overview of enriched GO terms of co-expression cluster (gene-centric) from all 5 datasets and MCL clusters predicted to contain the corresponding query probeset in different datasets.
Query gene
Gene-centric co-expression cluster of undefined
Query gene annotation
C.sinensis
Fruit
Leaf
Stress
Citrus
Global overview
MCL clusters
Co-expressed genes lists (Top 100 HRR) and HRR in corresponding conditions
Click on rows containing probesets of interests to launch a new gene-centric co-expression query of selected probeset
Hide/show additional information on co-expressed genes
GO functional enrichment
Tissue/condition specificity
Over-represented GO terms within co-expressed genes lists (Top 100 rank)
GO term(s) are considered enriched if the FDR<0.05 and having at least 2 genes in input gene lists
Hide/show input list
Global heatmap overview of GO over-representation across all datasets
Legend
1: Citrus, 2: C.sinensis, 3: Fruit, 4: Leaf, 5: Stress
Values are expressed as -log10(FDR) and '0' in columns represents no enrichment
BP
MF
CC
Lists of MCL clusters inferred from different datasets predicted to contain the corresponding probeset of interest
Click on respective columns to browse MCL clusters in different conditions with probesets as guide
Tissue/condition expression specificity of query gene and co-expression cluster
Cumulative expression specificity index (cluster)
query gene
rank1
rank2
rank3
rank4
rank5
Hide or show tissue/condition specificity table

Gene-centric co-expressed cluster page: Gene-centric co-expression of CsLOB1in *C.sinensis* condition as example

(1) The query gene (general) tab contains a table of the probe, symbol, predicted function based on BLAST analysis against Arabidopsis and *Citrus sinensis* using probeset consensus, predicted protein localization and best Arabidopsis and *C. sinensis* match of the query gene annotation of the probesets of interest. In this example, the screenshot contains query gene annotation of CsLOB1.

(2) The co-expressed genes tab labelled as *C. sinensis* (and others i.e. "citrus", "fruit") contains a table of co-expressed genes lists (Top 100 HRR) and HRR in corresponding condition and relevant information in the order or rank, co-expressed target of query, symbol, PCC value, and HRR in corresponding condition and across all other. In this example, screenshots of the top 10 rank co-expressed target with CsLOB1 is shown. Co-expression stability of HRR was shown in Csin (the condition of interests) as well as other 4 condition in order of "citrus", "fruit", "stress" and "leaf". *Click hide/show additional information on co-expressed genes to update the table to contain the predicted function information seen in. Click on rows containing probesets of interests to launch a new gene-centric co-expression query of selected probeset.*

(3) The GO functional enrichment tab contains a table of over-represented GO terms within co-expressed genes lists (Top 100 rank). GO term(s) are considered enriched if the FDR<0.05 and having at least 2 genes in input gene lists. *Clicking hide/show input lists will update the table to contain relevant probesets information enriched with the respective GO ID.*

(4) The tissue/condition specificity tab contains an interactive graph of the tissue/condition co-expression specificity of query gene and co-expression cluster. In this example, the cumulative expression specificity index (cESI) of the query (colored in blue) and the top 5 co-expressed genes (coloured in order of dark blue, green, red, cyan and purple) are shown as a stacked chart. *Simply hover the mouse cursor across the chart and a tooltip will appear showing the underlying condition (sample ID) and cESI value. Clicking the bars on the chart will update the panel underneath the chart to show a subset (user-selected condition of interests) of the tissue/condition specificity table. To view all relevant cESI scores, click the hide/show tissue/condition specificity table.* The tissue/condition specificity table contains the relevant sample and experiment ID, description, species, organ, treatment and cESI values of query and top 5 co-expressed genes.

(5) The raw expression data tab contains normalised (log2) gene expression data co-expressed genes (Top 100 rank) of query. *Mouse-over to view basic information such as gene information, intensity value under respective conditions, and detailed experimental description underlying the gene(s) of interests. To view a heatmap illustration of the normalised gene expression data click the hide raw gene expression (Heatmap overview) checkbox.*

(6) The global overview tab contains an overview of GO (Biological processes, molecular function and cellular component) enrichment of co-expressed genes across datasets are shown as an interactive heatmap table containing GO ID, type and description followed by enrichment value [expressed as -log10(P-value)] shown as a heatmap colours and raw values across "csin", "citrus", "fruit", "stress" and "leaf" datasets (if available).

(7) The MCL clusters tab contains a table with MCL clusters inferred from different datasets predicted to contain the corresponding probeset of interest. *Click on respective columns to browse MCL clusters in different conditions with probesets as guide.*

1

Gene-centric co-expression cluster of Cit.37210.1.S1_at

Query gene annotation

**Probe**	Cit.37210.1.S1_at
**Symbol**	LBD1
**Function_At**	LBD1 (LOB DOMAIN-CONTAINING PROTEIN 1)
**Function_Cs**	Putative uncharacterized protein Sb01g038650
**Psort**	NUC,CHL,VAC
**Best At match**	AT1G07900
**Best Cs match**	Cs7g27640.1

2

Co-expressed genes lists (Top 100 HRR) and HRR in corresponding conditions

Click on rows containing probesets of interests to launch a new gene-centric co-expression query of selected probeset

☑ Hide/show additional information on co-expressed genes

Rank	Target	symbol	PCC	Csin	Citrus	Fruit	Stress	Leaf
1	Cit.35190.1.S1_at	LBD11	1.00	1	1	1	1	2
2	Cit.38459.1.S1_at	NA	0.96	8	706	17651	116	17
3	Cit.19147.1.S1_at	NA	0.96	9	3872	15919	475	126
4	Cit.5370.1.S1_s_at	invertase	0.95	10	3	307	4	23
4	Cit.24058.1.S1_s_at	NA	0.97	10	5557	11044	974	29
5	Cit.30858.1.S1_at	EXPA4	0.97	11	3293	11430	297	10
5	Cit.3554.1.S1_s_at	ATCEL5 ATGH9B4CEL5	0.97	11	11948	24709	1029	46
5	Cit.20041.1.S1_at	NA	0.95	11	178	7633	19	11
6	Cit.14005.1.S1_at	EXPA4	0.97	12	4519	13944	408	11
6	Cit.12077.1.S1_at	NA	0.94	12	14687	21025	3785	13
7	Cit.37306.1.S1_at	zinc finger	0.94	13	1135	4381	151	87
8	Cit.14005.1.S1_s_at	EXPA4	0.96	14	10718	17031	1058	11
9	Cit.10235.1.S1_at	photoassimilate-responsive	0.96	16	884	6812	62	4
10	Cit.19513.1.S1_at	BCS1	0.93	18	5176	14721	388	99
10	Cit.32625.1.S1_s_at	photoassimilate-responsive	0.94	18	1777	8879	202	18
10	Cit.12550.1.S1_at	NA	0.94	18	6506	12307	630	25

Function
Function_At: LBD11 (LOB DOMAIN-CONTAINING PROTEIN 11) Function_Cs: Putative uncharacterized protein Sb01g038650 Psort: NUC,CHL,VAC Best At match: AT2G28500 Best Cs match: Cs7g27640.1

3

Over-represented GO terms within co-expressed genes lists (Top 100 rank)

GO term(s) are considered enriched if the FDR<0.05 and having at least 2 genes in input gene lists

☐ Hide/show input list

GO ID	GO type	GO description	# in input	# in background	P-val	FDR
GO:0071555	BP	cell wall organization	7	95	2.27E-07	5.8E-05
GO:0071554	BP	cell wall organization or biogenesis	8	95	7.59E-07	0.000129
GO:0005976	BP	polysaccharide metabolic process	8	95	3.98E-05	0.00371
GO:0009664	BP	plant-type cell wall organization	4	95	4.42E-05	0.00371
GO:0005975	BP	carbohydrate metabolic process	12	95	0.000176	0.0105
GO:0071669	BP	plant-type cell wall organization or biogenesis	4	95	0.000205	0.0105
GO:0006073	BP	cellular glucan metabolic process	6	95	0.000462	0.0196
GO:0044042	BP	glucan metabolic process	6	95	0.000462	0.0196
GO:0044264	BP	cellular polysaccharide metabolic process	6	95	0.000545	0.0214
GO:0005985	BP	sucrose metabolic process	5	95	0.00108	0.0306
GO:0005982	BP	starch metabolic process	5	95	0.00135	0.0331
GO:0016137	BP	glycoside metabolic process	5	95	0.00191	0.0407
GO:0005576	CC	extracellular region	11	95	2.53E-09	1.29E-06
GO:0031982	CC	vesicle	12	95	0.000723	0.0217
GO:0031410	CC	cytoplasmic vesicle	12	95	0.000709	0.0217
GO:0016023	CC	cytoplasmic membrane-bounded vesicle	12	95	0.000682	0.0217
GO:0031988	CC	membrane-bounded vesicle	12	95	0.000696	0.0217
GO:0016798	MF	hydrolase activity, acting on glycosyl bonds	8	95	1.22E-05	0.00156
GO:0004553	MF	hydrolase activity, hydrolyzing O-glycosyl compounds	7	95	5.09E-05	0.00371
GO:0008810	MF	cellulase activity	2	95	0.000196	0.0105
GO:0030570	MF	pectate lyase activity	2	95	0.00149	0.0331
GO:0016837	MF	carbon-oxygen lyase activity, acting on polysaccharides	2	95	0.00149	0.0331

4

Tissue/condition expression specificity of query gene and co-expression cluster

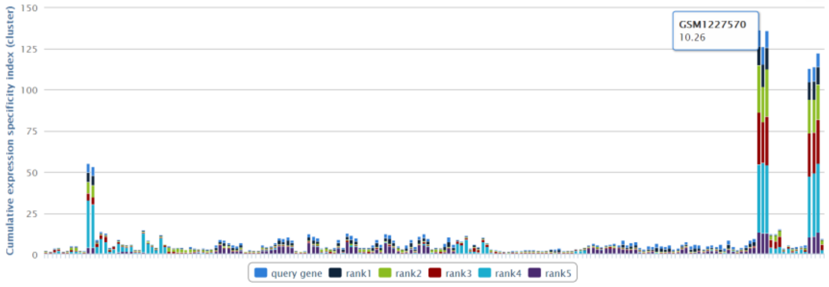

SamID	ExpID	Sample description	Species	Organ	Treatment	query gene	rank1	rank2	rank3	rank4	rank5
GSM1227570	GSE50741	Sweet orange leaves inoculated with Xcc306 wild type (WT) at 120 hpi	Csin	Leaf	Biotic	10.26	12.9	28.64	29.92	40.93	12.57

☑ Hide or show tissue/condition specificity table

SamID	ExpID	Sample description	Species	Organ	Treatment	query gene	rank1	rank2	rank3	rank4	rank5
GSM272741	GSE10798	water infiltrated leaves at 6h_biol replicate 1	Csin	Leaf	Abiotic	0.12	0.35	0.42	0.55	0.13	0.09
GSM272742	GSE10798	water infiltrated leaves at 6h_biol replicate 2	Csin	Leaf	Abiotic	0.09	0.3	0.25	0.31	0.24	0.1
GSM272743	GSE10798	water infiltrated leaves at 48h_biol replicate 1	Csin	Leaf	Abiotic	0.34	0.29	0.32	1.13	0.81	0.12
GSM272744	GSE10798	water infiltrated leaves at 48h_biol replicate 2	Csin	Leaf	Abiotic	0.24	0.25	0.27	1.31	1.33	0.16
GSM272745	GSE10798	Xaa infiltrated leaves at 6h_biol replicate 1	Csin	Leaf	Biotic	0.11	0.31	0.36	0.33	0.16	0.07
GSM272746	GSE10798	Xaa infiltrated leaves at 6h_biol replicate 2	Csin	Leaf	Biotic	0.12	0.27	0.62	0.53	0.16	0.12
GSM272747	GSE10798	Xaa infiltrated leaves at 48h_biol replicate 1	Csin	Leaf	Biotic	0.66	0.46	1.67	0.74	1.15	0.08
GSM272748	GSE10798	Xaa infiltrated leaves at 48h_biol replicate 2	Csin	Leaf	Biotic	0.67	0.49	1.76	0.56	0.98	0.14
GSM272749	GSE10798	Xac infiltrated leaves at 6h_biol replicate 1	Csin	Leaf	Biotic	0.12	0.34	0.37	0.43	0.33	0.14
GSM272750	GSE10798	Xac infiltrated leaves at 6h_biol replicate 2	Csin	Leaf	Biotic	0.14	0.26	0.37	0.45	0.23	0.1
GSM272751	GSE10798	Xac infiltrated leaves at 48h_biol replicate 1	Csin	Leaf	Biotic	5.21	5.6	7	4.5	28.39	3.71
GSM272752	GSE10798	Xac infiltrated leaves at 48h_biol replicate 2	Csin	Leaf	Biotic	5.1	5.87	7.13	4.36	26.41	3.62
GSM734400	GSE29633	leaves_mock-inoculated control, biological rep1	Csin	Leaf	Healthy	1.03	1.01	0.39	1.83	4.04	0.3
GSM734401	GSE29633	leaves_mock-inoculated control, biological rep2	Csin	Leaf	Healthy	0.93	0.72	0.39	2.2	8.81	0.26
GSM734402	GSE29633	leaves_mock-inoculated control, biological rep3	Csin	Leaf	Healthy	0.94	0.65	0.39	3.32	6.68	0.4

5

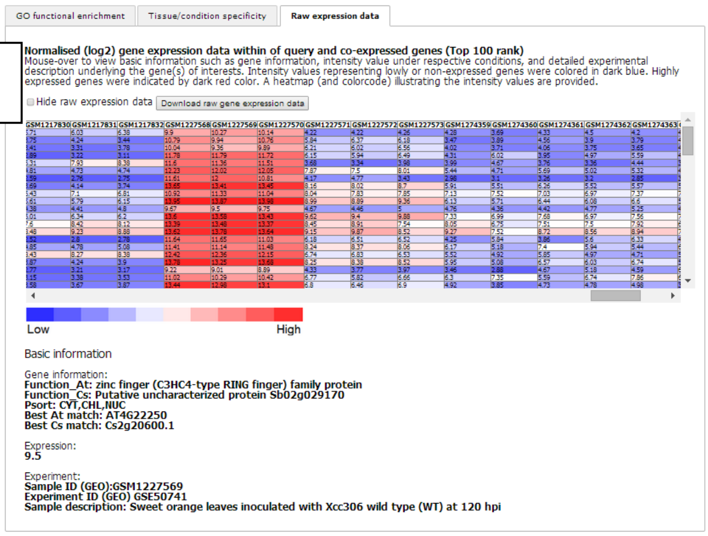

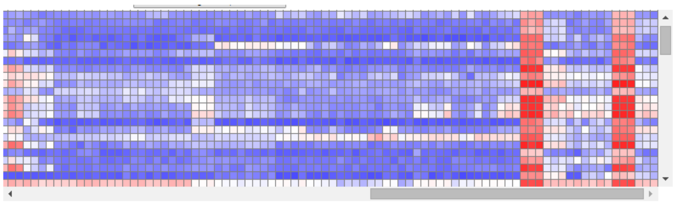

6

Global heatmap overview of GO over-representation across all datasets

Legend

1: Citrus, 2: C.sinensis, 3: Fruit, 4: Leaf, 5: Stress

Values are expressed as -log10(FDR) and '0' in columns represents no enrichment

GO ID	GO type	GO description	2	3	4	5	1	Csin	Fruit	leaf	Stress	Citrus
GO:0005975	BP	carbohydrate metabolic process						1.98	0	0	0	0
GO:0005976	BP	polysaccharide metabolic process						2.43	0	1.57	0	0
GO:0005982	BP	starch metabolic process						1.48	0	0	0	0
GO:0005984	BP	disaccharide metabolic process						1.48	0	0	0	0
GO:0005985	BP	sucrose metabolic process						1.51	0	0	0	0
GO:0006006	BP	glucose metabolic process						0	0	0	0	1.35
GO:0006073	BP	cellular glucan metabolic process						1.71	0	1.37	0	0
GO:0006091	BP	generation of precursor metabolites and energy						0	0	0	0	1.87
GO:0006098	BP	pentose-phosphate shunt						0	0	0	0	1.31
GO:0006119	BP	oxidative phosphorylation						0	0	0	0	1.73
GO:0006259	BP	DNA metabolic process						0	0	1.86	0	0
GO:0006260	BP	DNA replication						0	0	4.56	0	0
GO:0006261	BP	DNA-dependent DNA replication						0	0	8.74	1.51	0
GO:0006270	BP	DNA-dependent DNA replication initiation						0	0	8.58	2.83	0
GO:0006323	BP	DNA packaging						0	0	2.29	0	0
GO:0006333	BP	chromatin assembly or disassembly						0	0	1.33	0	0
GO:0006334	BP	nucleosome assembly						0	0	1.59	0	0
GO:0006575	BP	cellular amino acid derivative metabolic process						0	2.5	0	0	0
GO:0006732	BP	coenzyme metabolic process						0	0	0	0	1.85
GO:0006733	BP	oxidoreduction coenzyme metabolic process						0	0	0	0	1.85
GO:0006739	BP	NADP metabolic process						0	0	0	0	1.31
GO:0006740	BP	NADPH regeneration						0	0	0	0	1.31
GO:0006818	BP	hydrogen transport						0	0	0	0	1.31
GO:0007047	BP	cellular cell wall organization						0	0	2.15	0	0

7

Lists of MCL clusters inferred from different datasets predicted to contain the corresponding probeset of interest

Click on respective columns to browse MCL clusters in different conditions with probesets as guide

Probeset	Csin	Fruit	Leaf	Stress	Citrus
Cit.37210.1.S1_at	6	29	71	225	68

MCL cluster information page: Overview

The MCL cluster information page contains tabs for visualisation of global (and local) cluster interactions using CytoscapeWeb, tabular information on nodes (genes) and edges (weighted HRR) within the cluster, over-representation of GO categories of cluster members, a cluster tissue/condition specificity graph, and a table of significantly connected clusters in the predefined conditions [e.g. 'Citrus (condition-indepedent)',

'Csin(condition-specific)', 'Fruit(condition-specific)', 'Leaf(condition-specific)' and 'Stress(condition specific)'].

MCL cluster information

Explore MCL cluster information page containing tabs for visualisation of global (and local) cluster interactions using CytoscapeWeb, tabular information on nodes (genes) and edges (weighted HRR) within cluster, over-representation of GO catogories of cluster members, cluster tissue/condition specificity graph and significantly connected cluster table in the predefined conditions [e.g. 'Citrus (condition-indepedent)', 'Csin(condition-specific)', 'Fruit(condition-specific)', 'Leaf(condition-specific)' and 'Stress(condition-specific)'].

Global overview | Cluster nodes | Cluster edges | GO enrichment | Cluster ESI | Connected clusters

Gene co-expression cluster visualization
Mouse-over nodes to view gene function and network neighbourhood.
Right-click to view network at HRR cutoffs of 10, 20 and 30.
Double-click on nodes to view selected nodes network neighbourhood below cluster network.

List of co-expressed genes and functional annotation of selected node in cluster

Global overview | **Cluster nodes** | Cluster edges | GO enrichment | Cluster ESI | Connected clusters

List of all nodes within cluster
☐ Hide/show additional information on genes
Click on probeset ID to view the selected probeset network neighbourhood in a new window.

Global overview | Cluster nodes | **Cluster edges** | GO enrichment | Cluster ESI | Connected clusters

Lists of all edge relationships within this cluster

Global overview | Cluster nodes | Cluster edges | **GO enrichment** | Cluster ESI | Connected clusters

Over-represented GO terms within clusters' member genes
GO term(s) are considered enriched if the FDR<0.05 and having at least 2 genes in input gene lists ☐ Hide/show input lists

Global overview | Cluster nodes | Cluster edges | GO enrichment | **Cluster ESI** | Connected clusters

Mode of tissue/condition expression speicificity of clusters' member genes

Cluster tissue/condition expression specificity

Fraction, % is the # of probesets having ESI>1/total # probesets in cluster
☐ Hide or show tissue/condition specificity table

Global overview | Cluster nodes | Cluster edges | GO enrichment | Cluster ESI | **Connected clusters**

List of top 10 connected clusters with query cluster predicted to contain enriched GO term
Mouse-over rows to preview selected connected cluster's enriched GO terms in table below

Preview connected cluster, ☐ enriched GO terms

MCL cluster information page: Citrus MCL cluster 14 putatively involved in citrus peel isoprenoid and phenylpropanoid metabolism as an example

(1) The global overview tab contains the gene co-expression cluster visualization tool powered by CytoscapeWeb interface allowed users to visualise the relevant MCL inferred network and browse nodes within the cluster. *Mouse-over nodes to view gene function and select network neighbourhood. Right-click to view network at HRR cut-offs of 10, 20 and 30. Double-click on nodes (to view selected nodes network neighbourhood below cluster network. In this example, mouse over the node annotated as zinc finger (Cit.7748.1.S1_at). This highlights the first neighbours of the latter and popups the tooltip containing query gene annotation. Double click the node will update the panel below and lists functional annotation of co-expressed genes (first neighbours).*

(2) The cluster nodes tab contains the table list of all nodes within cluster. *Clicking hide/show additional information on genes expands the table to contain condition, cluster, probeset, symbol, function, best At and Cs match. Click on probeset ID to view the selected probeset network neighbourhood in a new window [see (1)].*

(3) The cluster lists tab contains lists of all edge relationships within this cluster in order of condition, cluster ID, interacting (co-expressed) Probeset 1 – 2 and weight. *Click on probeset ID to view the selected probeset network neighbourhood in a new window [see (1)].*

(4) The GO enrichment tab contains a table of over-represented GO terms within clusters' member genes. GO term(s) are considered enriched if the FDR<0.05 and having at least 2 genes in input gene lists. *Clicking hide/show input lists will update the table to contain relevant probesets information enriched with the respective GO ID.*

(5) The cluster ESI tab contains an interactive graph of the mode of tissue/condition co-expression specificity of clusters' member genes. The fraction, expressed as % (the # of probesets having ESI>1/total # probesets in cluster) is the expression specificity of module members in a particular tissue or condition (and across all arrays) with an expression specificity index above 1. *Simply hover the mouse cursor across the chart and a tooltip will appear showing the underlying condition (sample ID) and fraction value. Clicking the bars on the chart will update the panel underneath the chart to show a subset (user-selected condition of interests) of the tissue/condition specificity table. To view all relevant cESI scores, click the hide/show tissue/condition specificity table.* The tissue/condition specificity table contains the relevant sample and experiment ID, description, species, organ, treatment and fraction values of clusters' member genes.

(6) The connected clusters tab contains a list of top 10 connected clusters with query cluster predicted to contain enriched GO term. *Mouse-over rows to preview selected connected cluster's enriched GO terms, description and enrichment values (FDR) in a table.*

(7) The raw expression data tab contains normalised (log2) gene expression data of underlying MCL inferred co-expression cluster. *Mouse-over to view basic information such as gene information, intensity value under respective conditions, and detailed experimental description underlying the gene(s) of interests. To view a heatmap illustration of the normalised gene expression data click the hide raw gene expression (Heatmap overview) checkbox.*

1

Gene co-expression cluster visualization

Mouse-over nodes to view gene function and network neighbourhood.
Right-click to view network at HRR cutoffs of 10, 20 and 30.
Double-click on nodes to view selected nodes network neighbourhood below cluster network.

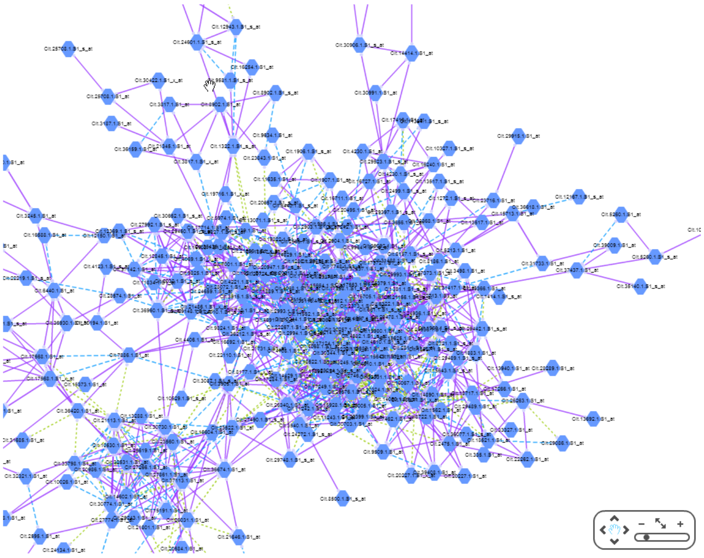

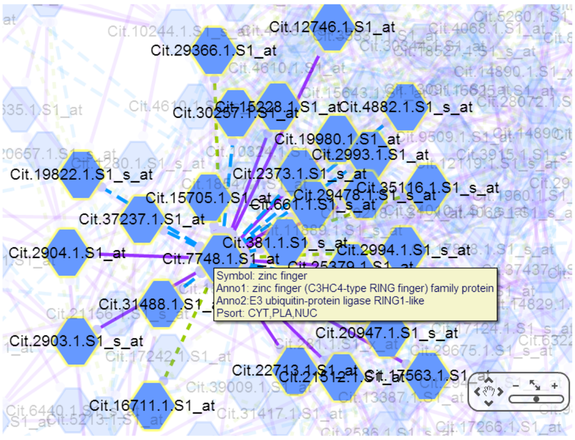

List of co-expressed genes and functional annotation of selected node in cluster

Probeset ID	Target	Weight	Symbol	Function	Condition
Cit.7748.1.S1_at	Cit.22713.1.S1_at	0.2	NA	Function_At: NA<br>Function_Cs: 3-hydroxybenzoate 6-hydroxylase<br>Psort: NUC,CHL,MIT<br>Best At match: 0<br>Best Cs match: Cs4g20530.1	Citrus
Cit.7748.1.S1_at	Cit.17372.1.S1_at	0.0667	HYD1	Function_At: HYD1 (HYDRA1)<br>Function_Cs: Phenylalkylamine Ca2+ antagonist (Emopamil) binding protein<br>Psort: NUC,CSK,MIT<br>Best At match: AT1G20050<br>Best Cs match: Cs5g21830.1	Citrus
Cit.7748.1.S1_at	Cit.12746.1.S1_at	0.2	methyltransferase	Function_At: O-methyltransferase family 2 protein<br>Function_Cs: Putative orcinol O-methyltransferase (Fragment)<br>Psort: CHL,MIT,PLA<br>Best At match: AT4G35160<br>Best Cs match: orange1.1t01353.1	Citrus
Cit.7748.1.S1_at	Cit.19822.1.S1_s_at	0.0667	FLO2	Function_At: AP2 (APETALA 2)<br>Function_Cs: Putative transcription factor AP2 family protein, 3'-partial (Fragment)<br>Psort: NUC,PLA,MIT<br>Best At match: AT4G36920<br>Best Cs match: Cs6g04120.1	Citrus
Cit.7748.1.S1_at	Cit.29478.1.S1_s_at	0.2	CYP82C4	Function_At: CYP82C4<br>Function_Cs: Cytochrome P450 82C4<br>Psort: END,CHL,NUC<br>Best At match: AT4G31940<br>Best Cs match: Cs1g10540.1	Citrus
Cit.7748.1.S1_at	Cit.11552.1.S1_s_at	0.0667	CYP71B23	Function_At: CYP71B23<br>Function_Cs: Premnaspirodiene oxygenase<br>Psort: END,PLA,CHL<br>Best At match: [illegible]	Citrus

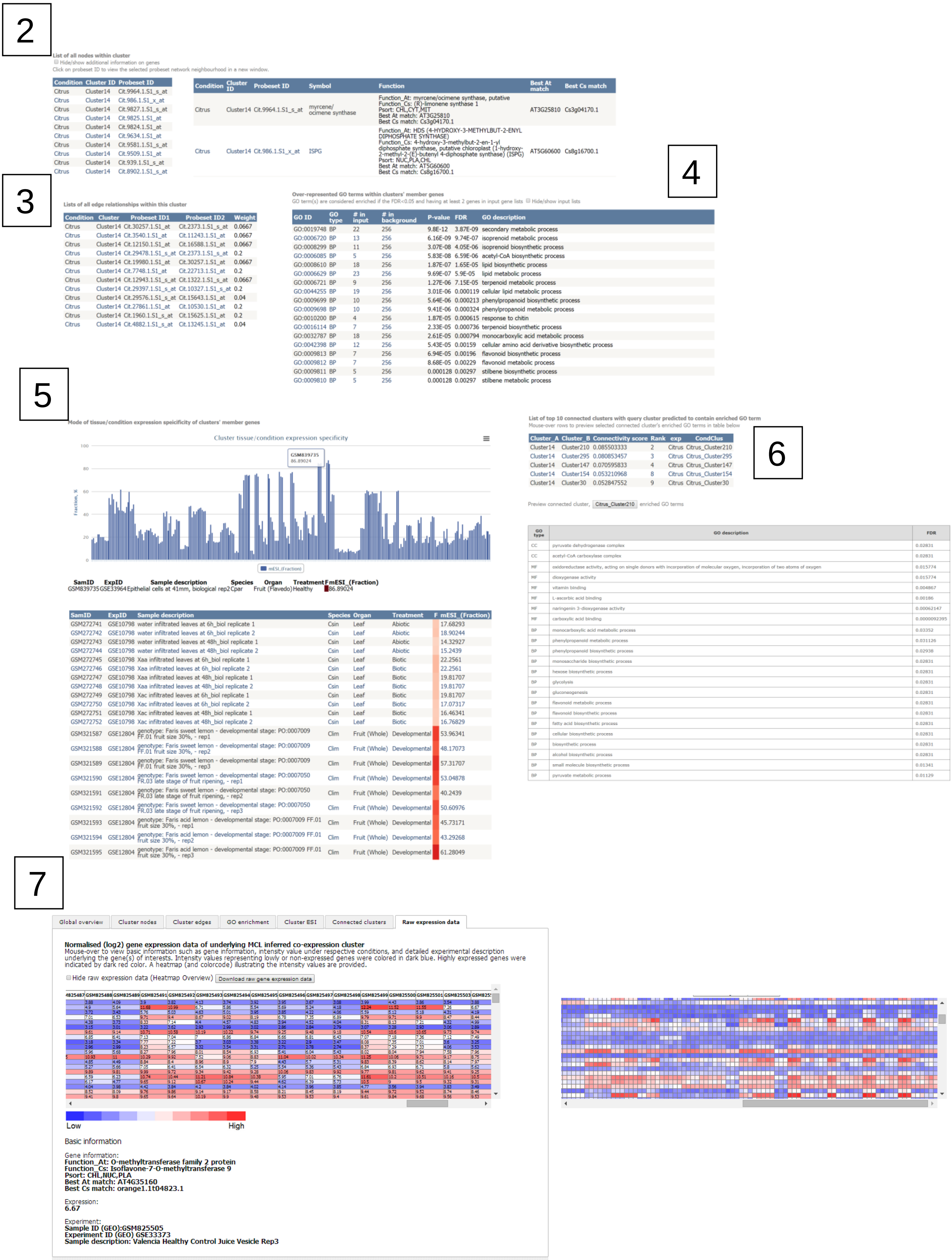
2
List of all nodes within cluster
Hide/show additional information on genes
Click on probeset ID to view the selected probeset network neighbourhood in a new window.
Condition Cluster ID Probeset ID
Citrus Cluster14 Cit.9964.1.S1_s_at
Citrus Cluster14 Cit.986.1.S1_x_at
Citrus Cluster14 Cit.9827.1.S1_s_at
Citrus Cluster14 Cit.9825.1.S1_at
Citrus Cluster14 Cit.9824.1.S1_at
Citrus Cluster14 Cit.9634.1.S1_at
Citrus Cluster14 Cit.9581.1.S1_s_at
Citrus Cluster14 Cit.9509.1.S1_at
Citrus Cluster14 Cit.939.1.S1_s_at
Citrus Cluster14 Cit.8902.1.S1_s_at
Condition Cluster ID Probeset ID Symbol Function Best At match Best Cs match
Citrus Cluster14 Cit.9964.1.S1_s_at myrcene/ ocimene synthase
Function_At: myrcene/ocimene synthase, putative
Function_Cs: (R)-limonene synthase 1
Psort: CHL,CYT,MIT
Best At match: AT3G25810
Best Cs match: Cs3g04170.1
AT3G25810 Cs3g04170.1
Citrus Cluster14 Cit.986.1.S1_x_at ISPG
Function_At: HDS (4-HYDROXY-3-METHYLBUT-2-ENYL DIPHOSPHATE SYNTHASE)
Function_Cs: 4-hydroxy-3-methylbut-2-en-1-yl diphosphate synthase, putative chloroplast (1-hydroxy-2-methyl-2-(E)-butenyl 4-diphosphate synthase) (ISPG)
Psort: NUC,PLA,CHL
Best At match: AT5G60600
Best Cs match: Cs8g16700.1
AT5G60600 Cs8g16700.1
3
Lists of all edge relationships within this cluster
Condition Cluster Probeset ID1 Probeset ID2 Weight
Citrus Cluster14 Cit.30257.1.S1_s_at Cit.2373.1.S1_s_at 0.0667
Citrus Cluster14 Cit.3540.1.S1_at Cit.11243.1.S1_at 0.0667
Citrus Cluster14 Cit.12150.1.S1_at Cit.16588.1.S1_at 0.0667
Citrus Cluster14 Cit.29478.1.S1_s_at Cit.2373.1.S1_s_at 0.2
Citrus Cluster14 Cit.19980.1.S1_at Cit.30257.1.S1_at 0.0667
Citrus Cluster14 Cit.7748.1.S1_at Cit.22713.1.S1_at 0.2
Citrus Cluster14 Cit.12943.1.S1_s_at Cit.1322.1.S1_s_at 0.0667
Citrus Cluster14 Cit.29397.1.S1_s_at Cit.10327.1.S1_s_at 0.2
Citrus Cluster14 Cit.29576.1.S1_s_at Cit.15643.1.S1_at 0.04
Citrus Cluster14 Cit.27861.1.S1_at Cit.10530.1.S1_at 0.2
Citrus Cluster14 Cit.1960.1.S1_s_at Cit.15625.1.S1_at 0.2
Citrus Cluster14 Cit.4882.1.S1_s_at Cit.13245.1.S1_at 0.04
4
Over-represented GO terms within clusters' member genes
GO term(s) are considered enriched if the FDR<0.05 and having at least 2 genes in input gene lists
Hide/show input lists
GO ID GO type # in input # in background P-value FDR GO description
GO:0019748 BP 22 256 9.8E-12 3.87E-09 secondary metabolic process
GO:0006720 BP 13 256 6.16E-09 9.74E-07 isoprenoid metabolic process
GO:0008299 BP 11 256 3.07E-08 4.05E-06 isoprenoid biosynthetic process
GO:0006085 BP 5 256 5.83E-08 6.59E-06 acetyl-CoA biosynthetic process
GO:0008610 BP 18 256 1.87E-07 1.65E-05 lipid biosynthetic process
GO:0006629 BP 23 256 9.69E-07 5.9E-05 lipid metabolic process
GO:0006721 BP 9 256 1.27E-06 7.15E-05 terpenoid metabolic process
GO:0044255 BP 19 256 3.01E-06 0.000119 cellular lipid metabolic process
GO:0009699 BP 10 256 5.64E-06 0.000213 phenylpropanoid biosynthetic process
GO:0009698 BP 10 256 9.41E-06 0.000324 phenylpropanoid metabolic process
GO:0010200 BP 4 256 1.87E-05 0.000615 response to chitin
GO:0016114 BP 7 256 2.33E-05 0.000736 terpenoid biosynthetic process
GO:0032787 BP 18 256 2.61E-05 0.000794 monocarboxylic acid metabolic process
GO:0042398 BP 12 256 5.43E-05 0.00159 cellular amino acid derivative biosynthetic process
GO:0009813 BP 7 256 6.94E-05 0.00196 flavonoid biosynthetic process
GO:0009812 BP 7 256 8.68E-05 0.00229 flavonoid metabolic process
GO:0009811 BP 5 256 0.000128 0.00297 stilbene biosynthetic process
GO:0009810 BP 5 256 0.000128 0.00297 stilbene metabolic process
5
Mode of tissue/condition expression specificity of clusters' member genes
Cluster tissue/condition expression specificity
GSM839735
86.89024
Fraction, %
mESI_(Fraction)
SamID ExpID Sample description Species Organ Treatment F mESI_(Fraction)
GSM839735 GSE33964 Epithelial cells at 41mm, biological rep2 Cpar Fruit (Flavedo) Healthy 86.89024
SamID ExpID Sample description Species Organ Treatment F mESI_(Fraction)
GSM272741 GSE10798 water infiltrated leaves at 6h_biol replicate 1 Csin Leaf Abiotic 17.68293
GSM272742 GSE10798 water infiltrated leaves at 6h_biol replicate 2 Csin Leaf Abiotic 18.90244
GSM272743 GSE10798 water infiltrated leaves at 48h_biol replicate 1 Csin Leaf Abiotic 14.32927
GSM272744 GSE10798 water infiltrated leaves at 48h_biol replicate 2 Csin Leaf Abiotic 15.2439
GSM272745 GSE10798 Xaa infiltrated leaves at 6h_biol replicate 1 Csin Leaf Biotic 22.2561
GSM272746 GSE10798 Xaa infiltrated leaves at 6h_biol replicate 2 Csin Leaf Biotic 22.2561
GSM272747 GSE10798 Xaa infiltrated leaves at 48h_biol replicate 1 Csin Leaf Biotic 19.81707
GSM272748 GSE10798 Xaa infiltrated leaves at 48h_biol replicate 2 Csin Leaf Biotic 19.81707
GSM272749 GSE10798 Xac infiltrated leaves at 6h_biol replicate 1 Csin Leaf Biotic 19.81707
GSM272750 GSE10798 Xac infiltrated leaves at 6h_biol replicate 2 Csin Leaf Biotic 17.07317
GSM272751 GSE10798 Xac infiltrated leaves at 48h_biol replicate 1 Csin Leaf Biotic 16.46341
GSM272752 GSE10798 Xac infiltrated leaves at 48h_biol replicate 2 Csin Leaf Biotic 16.76829
GSM321587 GSE12804 genotype: Faris sweet lemon - developmental stage: PO:0007009 FF.01 fruit size 30%, - rep1 Clim Fruit (Whole) Developmental 53.96341
GSM321588 GSE12804 genotype: Faris sweet lemon - developmental stage: PO:0007009 FF.01 fruit size 30%, - rep2 Clim Fruit (Whole) Developmental 48.17073
GSM321589 GSE12804 genotype: Faris sweet lemon - developmental stage: PO:0007009 FF.01 fruit size 30%, - rep3 Clim Fruit (Whole) Developmental 57.31707
GSM321590 GSE12804 genotype: Faris sweet lemon - developmental stage: PO:0007050 FR.03 late stage of fruit ripening, - rep1 Clim Fruit (Whole) Developmental 53.04878
GSM321591 GSE12804 genotype: Faris sweet lemon - developmental stage: PO:0007050 FR.03 late stage of fruit ripening, - rep2 Clim Fruit (Whole) Developmental 40.2439
GSM321592 GSE12804 genotype: Faris sweet lemon - developmental stage: PO:0007050 FR.03 late stage of fruit ripening, - rep3 Clim Fruit (Whole) Developmental 50.60976
GSM321593 GSE12804 genotype: Faris acid lemon - developmental stage: PO:0007009 FF.01 fruit size 30%, - rep1 Clim Fruit (Whole) Developmental 45.73171
GSM321594 GSE12804 genotype: Faris acid lemon - developmental stage: PO:0007009 FF.01 fruit size 30%, - rep2 Clim Fruit (Whole) Developmental 43.29268
GSM321595 GSE12804 genotype: Faris acid lemon - developmental stage: PO:0007009 FF.01 fruit size 30%, - rep3 Clim Fruit (Whole) Developmental 61.28049
6
List of top 10 connected clusters with query cluster predicted to contain enriched GO term
Mouse-over rows to preview selected connected cluster's enriched GO terms in table below
Cluster_A Cluster_B Connectivity score Rank exp CondClus
Cluster14 Cluster210 0.085503333 2 Citrus Citrus_Cluster210
Cluster14 Cluster295 0.080853457 3 Citrus Citrus_Cluster295
Cluster14 Cluster147 0.070595833 4 Citrus Citrus_Cluster147
Cluster14 Cluster154 0.053210968 8 Citrus Citrus_Cluster154
Cluster14 Cluster30 0.052847552 9 Citrus Citrus_Cluster30
Preview connected cluster, Citrus_Cluster210 enriched GO terms
GO type GO description FDR
CC pyruvate dehydrogenase complex 0.02831
CC acetyl-CoA carboxylase complex 0.02831
MF oxidoreductase activity, acting on single donors with incorporation of molecular oxygen, incorporation of two atoms of oxygen 0.015774
MF dioxygenase activity 0.015774
MF vitamin binding 0.004867
MF L-ascorbic acid binding 0.00186
MF naringenin 3-dioxygenase activity 0.00062147
MF carboxylic acid binding 0.0000092395
BP monocarboxylic acid metabolic process 0.03352
BP phenylpropanoid metabolic process 0.031126
BP phenylpropanoid biosynthetic process 0.02938
BP monosaccharide biosynthetic process 0.02831
BP hexose biosynthetic process 0.02831
BP glycolysis 0.02831
BP gluconeogenesis 0.02831
BP flavonoid metabolic process 0.02831
BP flavonoid biosynthetic process 0.02831
BP fatty acid biosynthetic process 0.02831
BP cellular biosynthetic process 0.02831
BP biosynthetic process 0.02831
BP alcohol biosynthetic process 0.02831
BP small molecule biosynthetic process 0.01341
BP pyruvate metabolic process 0.01129
7
Global overview
Cluster nodes
Cluster edges
GO enrichment
Cluster ESI
Connected clusters
Raw expression data
Normalised (log2) gene expression data of underlying MCL inferred co-expression cluster
Mouse-over to view basic information such as gene information, intensity value under respective conditions, and detailed experimental description underlying the gene(s) of interests. Intensity values representing lowly or non-expressed genes were colored in dark blue. Highly expressed genes were indicated by dark red color. A heatmap (and colorcode) illustrating the intensity values are provided.
Hide raw expression data (Heatmap Overview)
Download raw gene expression data
Low
High
Basic information
Gene information:
Function_At: O-methyltransferase family 2 protein
Function_Cs: Isoflavone-7-O-methyltransferase 9
Psort: CHL,NUC,PLA
Best At match: AT4G35160
Best Cs match: orange1.1t04823.1
Expression:
6.67
Experiment:
Sample ID (GEO):GSM825505
Experiment ID (GEO) GSE33373
Sample description: Valencia Healthy Control Juice Vesicle Rep3
